# Exploitation of nuclear functions by human rhinovirus, a cytoplasmic RNA virus

**DOI:** 10.1371/journal.ppat.1007277

**Published:** 2018-08-24

**Authors:** Dylan Flather, Joseph H. C. Nguyen, Bert L. Semler, Paul D. Gershon

**Affiliations:** 1 Department of Microbiology and Molecular Genetics, School of Medicine, University of California, Irvine, California, United States of America; 2 Center for Virus Research, University of California, Irvine, California, United States of America; 3 Department of Molecular Biology and Biochemistry, University of California, Irvine, California, United States of America; University of Wisconsin-Madison, UNITED STATES

## Abstract

Protein production, genomic RNA replication, and virion assembly during infection by picornaviruses like human rhinovirus and poliovirus take place in the cytoplasm of infected human cells, making them the quintessential cytoplasmic pathogens. However, a growing body of evidence suggests that picornavirus replication is promoted by a number of host proteins localized normally within the host cell nucleus. To systematically identify such nuclear proteins, we focused on those that appear to re-equilibrate from the nucleus to the cytoplasm during infection of HeLa cells with human rhinovirus via quantitative protein mass spectrometry. Our analysis revealed a highly selective re-equilibration of proteins with known mRNA splicing and transport-related functions over nuclear proteins of all other functional classes. The multifunctional splicing factor proline and glutamine rich (SFPQ) was identified as one such protein. We found that SFPQ is targeted for proteolysis within the nucleus by viral proteinase 3CD/3C, and a fragment of SFPQ was shown to migrate to the cytoplasm at mid-to-late times of infection. Cells knocked down for SFPQ expression showed significantly reduced rhinovirus titers, viral protein production, and viral RNA accumulation, consistent with SFPQ being a pro-viral factor. The SFPQ fragment that moved into the cytoplasm was able to bind rhinovirus RNA either directly or indirectly. We propose that the truncated form of SFPQ promotes viral RNA stability or replication, or virion morphogenesis. More broadly, our findings reveal dramatic changes in protein compartmentalization during human rhinovirus infection, allowing the virus to systematically hijack the functions of proteins not normally found at its cytoplasmic site of replication.

## Author summary

We explored the dynamics of host cell protein relocalization from the nucleus to the cytoplasm during an infection by human rhinovirus using quantitative mass spectrometry, confocal imaging, and Western blot analysis. We discovered a highly selective re-equilibration of proteins with known mRNA splicing and transport-related functions, including splicing factor proline and glutamine rich (SFPQ). Using RNAi experiments and viral replication assays, we demonstrated that SFPQ is a pro-viral factor required for rhinovirus growth. Our studies provide new insights into how this cytoplasmic RNA virus is able to alter and hijack the functions of host proteins that normally reside in the nucleus.

## Introduction

Viruses of the *Picornaviridae* are characterized by a positive polarity, single-stranded RNA genome of 7–10 kb within a non-enveloped icosahedral capsid. The genome contains a single open reading frame flanked by a long (>500 nucleotide) 5’-noncoding region (NCR), a shorter 3’-NCR, and a 3’-terminal poly(A) tract. Although not unique to picornaviruses, another feature of these viruses is the use of a viral protein, VPg, to prime viral RNA synthesis. As a result, VPg is covalently linked to the 5’-terminus of the viral RNA and is the only viral protein known to be encapsidated. Being of mRNA polarity, the viral genome is translated immediately following virus attachment, uncoating, and release into the cell cytoplasm. The 5’-NCR contains extensive RNA secondary structure elements, including an internal ribosome entry site (IRES) which drives the cap-independent translation of the viral genome. The multiple stem-loop structures of the IRES interact with cellular proteins to recruit ribosomes, initiating the synthesis of a polyprotein which is processed by viral proteinases 2A and 3CD/3C to produce both incompletely processed (yet functional) polyprotein intermediates and mature viral proteins [1–3]. Viral RNA replication, like translation, occurs in the cytoplasm of infected cells and employs the newly synthesized RNA-dependent RNA polymerase, 3D, in concert with non-structural viral proteins and host proteins. Genome replication is promoted by elements of secondary structure present at the termini of both positive- and negative-stranded viral RNA. These structures serve as the interaction sites for cellular RNA-binding proteins that are thought to promote intermolecular architectures conducive to this process [4–6]. RNA synthesis initially yields RNA intermediates of negative polarity, which then serve as templates for the production of genomic RNA. Nascent RNA genomes can then serve as templates for further rounds of translation and RNA replication and, upon production of sufficient viral protein, are encapsidated to yield mature, infectious virions. The resulting viral progeny then exit the cell via lysis and/or non-lytic release within extracellular vesicles [7].

Rhinoviruses, members of the enterovirus genus within the *Picornaviridae* family, are the causative agents of a plurality of all human respiratory infections [8, 9]. Although most rhinovirus infections in healthy individuals cause relatively benign and self-limiting upper respiratory disease (i.e., the common cold), these infections can have more serious effects in some patient populations. For example, severe lower respiratory tract infections can occur in individuals with asthma, and acute exacerbations can arise in those with chronic obstructive pulmonary disease. In some instances, rhinovirus infection can also result in fatal pneumonia in the immunocompromised and the elderly [10–13]. Rhinoviruses are categorized into three species (A, B, and C) comprising over 150 genotypes with broad (>12%) sequence divergence in the VP1 capsid gene [14, 15]. Attempts to develop a vaccine have been hindered by the resulting high antigenic diversity which, in part, accounts for the lack of rhinovirus vaccine clinical trials since the 1970s [16–18]. Moreover, antiviral agents for the treatment or prevention of rhinovirus infection have shown toxicity, combined with limited efficacy [19, 20]. Although severe disease symptoms are not normally associated with human rhinovirus (HRV) infections, there is a very substantial economic burden associated with the common cold [21]. Insights into rhinovirus replication could provide opportunities for the development of effective antivirals and relief from the significant health and economic burden associated with these infections.

Despite extensive study, largely in the poliovirus model, aspects of the enterovirus replication cycle remain poorly understood. One key feature of enterovirus replication is an alteration in the bidirectional movement of biomolecules through the nuclear pore complex (NPC), a highly organized protein complex that regulates molecular traffic through the nuclear envelope (reviewed in [22]). During infection, nucleocytoplasmic trafficking is disrupted by viral proteinases, which target nucleoporin (Nup) proteins of the NPC, for degradation. “FG-Nups,” which occupy the central channel of the NPC are the primary targets of viral proteolysis. These FG-Nups contain phenylalanine-glycine (FG) repeats along with unstructured regions that serve as contact sites for the soluble transport receptors (called karyopherins) that ferry cargo biomolecules through the pore (reviewed in [23]). Experiments using recombinant proteinases *in vitro* or transfected proteinase expression constructs in cell culture have shown Nup62, Nup98, and Nup153 to be targeted by enterovirus proteinase 2A, and Nup62, Nup153, Nup214, and Nup358 to be targeted by proteinase 3CD/3C [24–30]. During virus infection, the two viral proteinases likely act synergistically to degrade these FG-Nups [31, 32]. Enterovirus-induced Nup cleavage leads to a breakdown in the conduit by which proteins translocate between cytoplasm and nucleus and dysregulation in the barrier function of the NPC, which together result in the mislocalization of nuclear proteins as the infectious cycle progresses [24, 25, 33].

Although cellular nucleic acid binding proteins promote RNA virus replication, their localization within the cell may not be optimal for exploitation by the virus. However, targeting of the NPC leads to an alteration in the normal process of nucleocytoplasmic partitioning, effectively expanding the repertoire of protein functions available at sites where viral replication is carried out. Cellular proteins involved in enterovirus translation include nuclear shuttling proteins such as polypyrimidine tract binding protein 1 (PTBP1) and poly(r)C binding protein 2 (PCBP2). As a consequence of their normal cellular functions, these proteins are present in the cytoplasm of uninfected cells and therefore can be exploited immediately upon viral infection [34–36]. As the infectious cycle progresses, the NPC is degraded and proteins normally partitioning to the nucleus can be found in the cytoplasm where they can promote efficient viral RNA replication. One such protein is heterogeneous nuclear ribonucleoprotein C1/C2 (hnRNP C1/C2) [6, 37, 38]. This protein normally functions in pre-mRNA processing and is restricted to the nucleus [39, 40]. However, upon poliovirus infection, hnRNP C1/C2 redistributes to the cytoplasm where it promotes RNA replication, possibly through circularization of the negative strand intermediate RNA and recruitment of viral protein 3D [38, 41].

The experiments described in this study were based on the presumption that a more complete identification of nuclear proteins that relocalize to the cytoplasm during the infectious cycle would reveal novel factors involved in enterovirus replication. To identify these putative factors, we screened for proteins that become enriched in the cytoplasm of HeLa cells during infection with HRV16 (also known as rhinovirus A16), using quantitative protein mass spectrometry. We identified more than 30 host proteins whose steady-state abundance in the nucleus during rhinovirus infection clearly decreased, concomitant with a corresponding increase in cytoplasmic levels. One such protein was splicing factor proline and glutamine rich (SFPQ), a C-terminal cleavage fragment of which appeared in the nucleus at mid-times during infection then subsequently relocalized to the cytoplasm. This cleavage was independent of caspase activity, and a predicted HRV16 3CD/3C proteinase cleavage site was identified within SFPQ. Knockdown of SFPQ through an siRNA-mediated approach led to reductions in viral titer, protein production, and RNA accumulation, suggesting that SFPQ is a pro-viral factor for HRV16. We also show that the C-terminal fragment of SFPQ, in cytoplasmic extracts from infected cells, is able to bind HRV16 RNA. Overall, our analysis revealed major shifts in nuclear/cytoplasmic protein compartmentalization during rhinovirus infection of human cells, expanding our understanding of the importance of nuclear-resident proteins in the life cycle of a cytoplasmic RNA virus. In addition, we identify the multifunctional host cell nuclear protein SFPQ as a novel player in rhinovirus replication.

## Results

### HRV16 infection induces a coordinated redistribution of proteins into the cytoplasm of infected cells

**Fig 1:**
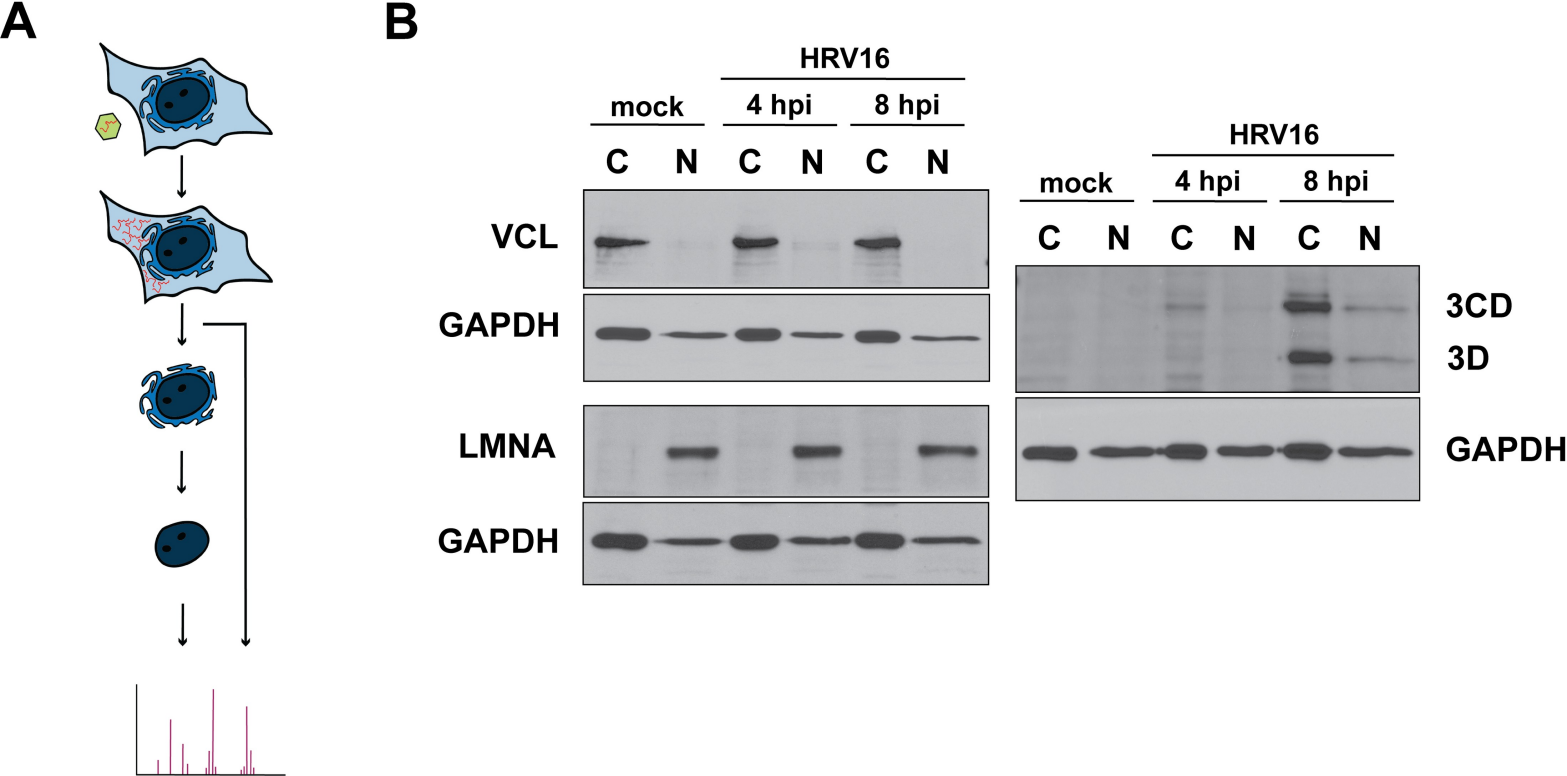
Subcellular fractionation of HRV16-infected HeLa cells for protein distribution analysis. (**A**) Mass spectrometry analysis of protein distribution. Mock- or HRV16-infected HeLa cells were separated into cytoplasmic and nuclear fractions, digested with trypsin, isotopically labeled, pooled, and subjected to nanoLC-MS/MS. (**B**) Fractionation of HeLa cells following mock, 4, or 8 hours post-HRV16 infection (hpi) was confirmed by Western blot analysis. Vinculin (VCL) was used as a cytoplasmic (C) marker protein and lamin A/C (LMNA) as a marker of the nucleus (N). As confirmation of a productive infection, fractions were assayed for the expression of HRV16 RNA-dependent RNA polymerase 3D and its precursor 3CD. Glyceraldehyde-3-phosphate dehydrogenase (GAPDH) was used as loading control.

To identify cellular proteins that re-equilibrate from the nucleus to the cytoplasm during HeLa cell infection with HRV16, we fractionated mock- and HRV16-infected cells at early (4 hours) and mid-to-late (8 hours) times of infection (Fig 1A) into their nuclear and cytoplasmic compartments by hypotonic swelling followed by Dounce homogenization and centrifugation [42]. Compartmental separation was confirmed by assaying the cytoskeletal protein vinculin and the nuclear matrix protein lamin A/C (Fig 1B). By 4 hours post-infection (hpi) viral proteinase 3CD was detected in cytoplasmic fractions, and by 8 hpi the viral RNA-dependent RNA polymerase 3D was present in both cytoplasmic and nuclear fractions, consistent with a productive infection of cells prior to fractionation (Fig 1B). The presence of HRV16 3CD within the nucleus has been reported previously [43].

Nuclear and cytoplasmic fractions from mock, 4 hr-, and 8 hr-infected cells were digested separately with trypsin, and tryptic peptides in the resulting six fractions were tagged with dimethyl isotope labels to distinguish mock, 4 hour, and 8 hour peptides. The differentially tagged peptides in three nuclear fractions were combined and subjected to strong cation exchange (SCX) chromatography followed by nanoLC-MS/MS of active SCX fractions. The SCX/nanoLC-MS/MS procedure was repeated for the three cytoplasmic fractions. Then the whole experiment was repeated in biological replicate, with isotope channel swapping to normalize for spectral interference. S1 Table gives an overview of database search and peak quantitation data for each of the resulting four MS datasets. Due to the limited impact of infection on protein redistribution at 4 hours post-infection, we focused our attention on the 8 hour time point. To screen for nucleus-to-cytoplasm re-equilibration, data were filtered in two ways, namely, at the protein level and peptide level.

At the protein level, 276 proteins showed an 8 hr:mock abundance ratio < 1.0 in at least one of the two nuclear datasets and > 1.0 in at least one of the two cytoplasmic datasets. These proteins are listed in S2 Table. A GO-term enrichment analysis was performed for the 276-accession set of S2 Table using, as a reference the combined, total proteomes of the two nuclear samples (a total of 6700 unique human accessions recognized by Panther, [44]). This analysis showed a remarkable enrichment in GO terms associated with mRNA splicing and transport (Table 1), with false discovery rate (FDR) values ranging as extreme as 10^−24^. These data clearly demonstrated a highly selective re-equilibration, from nucleus to cytoplasm, of proteins with known mRNA splicing and transport-related functions over nuclear proteins of all other functional classes. The transport-related GO terms further suggested that proteins may re-equilibrate to the cytoplasm as RNP complexes. In addition, the FDR for “mRNA splicing, via spliceosome” (GO:0000398) was ~17 orders of magnitude more significant than for “regulation of mRNA splicing, via spliceosome” (GO:0048024). Also of note was the enrichment for “catalytic step 2 spliceosome” (GO:0071013, final row of Table 1). Table 2 shows an abstraction of S2 Table, for those proteins detected in all four samples. These are considered the most highly-detectable (best characterized) of the nucleocytoplasmic re-equilibration set of S2 Table.

**Table 1:** Most highly enriched terms in the “GO complete” Panther database applicable to the 276-protein set of S2 Table, with respect to a reference database comprising the merged experimental HeLa nuclear proteomes (nuc1 and nuc2, S1 Table). *Fold enrichment*: Proportion of accessions with the given GO term in the 276-protein set / proportion in the 6700-member merged experimental nuclear reference proteome. *Raw P-value*: Raw statistical probability that the given fold-enrichment could have occurred by chance. *FDR* (false discovery rate): Proportion of decoy search results above raw P-value in paired target/decoy search. Terms are ranked by FDR: For “GO biological process” and GO molecular function”, all GO terms with an FDR > 1E-6 are shown. For “GO cellular component”, the threshold was 1E-10 since this was the approximate value for the GO term “nuclear part”, the compartment of origin.

Term code	Term descriptor: GO biological process (complete)	Fold enrichment	Raw P-value	FDR
GO:0008380	RNA splicing	5.3	1.06E-28	1.27E-24
GO:0006397	mRNA processing	4.89	4.65E-28	2.78E-24
GO:0000377	RNA splicing, via transesterification reactions with bulged adenosine as nucleophile	6.02	1.92E-27	5.73E-24
GO:0000375	RNA splicing, via transesterification reactions	5.97	2.79E-27	6.67E-24
GO:0000398	mRNA splicing, via spliceosome	6.02	1.92E-27	7.64E-24
GO:0016071	mRNA metabolic process	3.57	1.36E-21	2.72E-18
GO:0006396	RNA processing	2.88	7.87E-18	1.34E-14
GO:0050684	regulation of mRNA processing	6.77	1.40E-12	2.10E-09
GO:0051028	mRNA transport	5.25	1.59E-10	2.11E-07
GO:0048024	regulation of mRNA splicing, via spliceosome	7.35	2.37E-10	2.83E-07
GO:0050657	nucleic acid transport	4.49	1.29E-09	1.29E-06
GO:0050658	RNA transport	4.49	1.29E-09	1.40E-06
GO:0051236	establishment of RNA localization	4.43	1.68E-09	1.55E-06
GO:0006403	RNA localization	4.11	3.39E-09	2.90E-06
GO:1903311	regulation of mRNA metabolic process	3.69	4.16E-09	3.32E-06
GO:0006369	termination of RNA polymerase II transcription	6.9	4.78E-09	3.57E-06
GO:0006353	DNA-templated transcription, termination	5.54	5.33E-09	3.75E-06
GO:0015931	nucleobase-containing compound transport	4.14	5.92E-09	3.93E-06
GO:0043484	regulation of RNA splicing	5.47	6.30E-09	3.96E-06
GO:0071427	mRNA-containing ribonucleoprotein complex export from nucleus	5.3	1.03E-08	5.85E-06
GO:0006406	mRNA export from nucleus	5.3	1.03E-08	6.14E-06
GO:0006405	RNA export from nucleus	4.97	1.17E-08	6.33E-06
	**Term descriptor: GO molecular function (complete)**			
GO:0003723	RNA binding	2.18	1.62E-16	5.19E-13
GO:0003676	nucleic acid binding	1.59	2.84E-10	4.55E-07
	**Term descriptor: GO cellular component (complete)**			
GO:0044446	intracellular organelle part	1.32	1.29E-13	7.20E-11
GO:0071013	catalytic step 2 spliceosome	7.12	1.82E-13	7.63E-11

**Table 2:** Proteins whose abundance consistently increased in the cytoplasm while decreasing in the nucleus at 8 hr post-infection of HeLa cells with HRV16. This table is an abstraction of those proteins in S2 Table detected in all four datasets (‘Nuc1’, ‘Nuc2’, ‘Cyto1’, ‘Cyto2’). Delimited values ‘x/y/z’ refer to an 8hr:mock abundance ratio of x based on z tryptic peptide species, y of which tracked the direction (< 1 or > 1) of x. For additional details see S2 Table legend.

Accession	Description	Nuc1	Nuc2	Cyto1	Cyto2
ANXA2	Annexin A2	0.4317/21/23	0.1666/8/8	2.0875/14/16	1.2723/18/18
ANXA6	Annexin A6	0.132/7/7	0.0816/6/6	2.7491/6/6	1.3958/7/7
CD44	CD44 antigen	0.6967/1/4	0.2408/1/1	2.2396/4/4	1.269/4/5
COR1B	Coronin-1B	0.2592/5/5	0.0277/1/1	2.2832/3/3	2.1327/3/3
COR1C	Coronin-1C	0.1418/7/7	0.0432/3/3	25.9097/5/5	1.335/6/6
CPSF2	Cleavage and polyadenylation specificity factor subunit 2	0.2228/4/5	0.1982/2/2	2.005/1/1	4.5298/6/6
CSTF3	Cleavage stimulation factor subunit 3	0.5483/5/5	0.3508/2/2	10.25/1/1	7.0256/4/4
DHX9	ATP-dependent RNA helicase A	0.2869/32/34	0.2458/8/9	2.2561/8/9	1.6309/18/18
DREB	Drebrin	0.2133/9/9	0.1965/3/3	3.1428/3/3	1.2084/4/4
FLNA	Filamin-A	0.2329/56/59	0.044/22/22	3.6386/50/50	1.7896/50/52
FLNB	Filamin-B	0.266/36/37	0.0552/7/7	2.113/27/27	1.2044/38/43
HNRPL	Heterogeneous nuclear ribonucleoprotein L	0.4883/15/18	0.4397/4/4	4.6695/3/3	3.1403/6/6
HNRPM	Heterogeneous nuclear ribonucleoprotein M	0.1427/22/22	0.0963/10/10	3.1112/2/2	1.8638/9/9
MATR3	Matrin-3	0.2173/22/22	0.27/8/9	4.1396/3/3	2.0742/12/12
MTA2	Metastasis-associated protein MTA2	0.5761/14/16	0.3655/5/5	353.4703/4/4	1.7301/3/5
MTA3	Metastasis-associated protein MTA3	0.5277/6/6	0.3368/1/1	2.428/1/1	2.876/1/1
NONO	Non-POU domain-containing octamer-binding protein	0.2521/20/21	0.144/8/9	5.015/2/2	1.9679/5/5
OGT1	UDP-N-acetylglucosamine-peptide N-acetylglucosaminyltransferase 110 kDa subunit	0.5653/2/2	0.5655/1/1	47.43/1/1	26.1288/2/7
PABP2	Polyadenylate-binding protein 2	0.5335/7/7	0.2987/2/2	2.481/1/1	1.1887/3/3
PLEC	Plectin	0.2751/190/196	0.1086/72/74	4.4278/42/47	2.7159/77/79
PLOD3	Procollagen-lysine,2-oxoglutarate 5-dioxygenase 3	0.5699/10/11	0.4759/2/2	3.154/5/5	1.1828/8/11
PSPC1	Paraspeckle component 1	0.4501/10/12	0.2438/1/1	9.3991/3/3	2.0014/2/2
RBMX	RNA-binding motif protein, X chromosome	0.7064/13/15	0.285/1/1	6.884/1/1	3.2891/5/5
RBP2	E3 SUMO-protein ligase RanBP2	0.4485/31/38	0.3809/9/10	12.0081/4/4	1.5007/12/13
ROA1	Heterogeneous nuclear ribonucleoprotein A1	0.5264/14/15	0.0832/6/6	4.5871/5/5	1.3267/8/10
ROA2	Heterogeneous nuclear ribonucleoproteins A2/B1	0.543/17/19	0.3519/8/10	2.9374/7/7	2.2725/11/11
SAFB1	Scaffold attachment factor B1	0.5182/15/17	0.2757/6/6	6.238/4/4	3.6845/4/4
SF3A1	Splicing factor 3A subunit 1	0.5655/11/19	0.4464/8/9	3.1441/1/1	1.3703/11/12
SF3B1	Splicing factor 3B subunit 1	0.7293/23/31	0.5508/11/11	5.4408/3/3	1.9732/24/24
SFPQ	Splicing factor, proline- and glutamine-rich	0.372/26/26	0.0761/6/6	6.0995/8/8	2.5987/7/7
SPT6H	Transcription elongation factor SPT6	0.1824/20/20	0.3019/5/5	6.2192/3/3	1.2091/12/17
SPTN1	Spectrin alpha chain, non-erythrocytic 1	0.1479/82/83	0.0281/47/47	2.3006/26/28	1.4219/33/36
SYMPK	Symplekin	0.6877/4/4	0.2639/1/1	2.2432/1/1	1.6576/4/4
SYNE2	Nesprin-2	0.4411/8/12	0.3828/2/2	2.539/1/1	1.42/1/1

**Fig 2:**
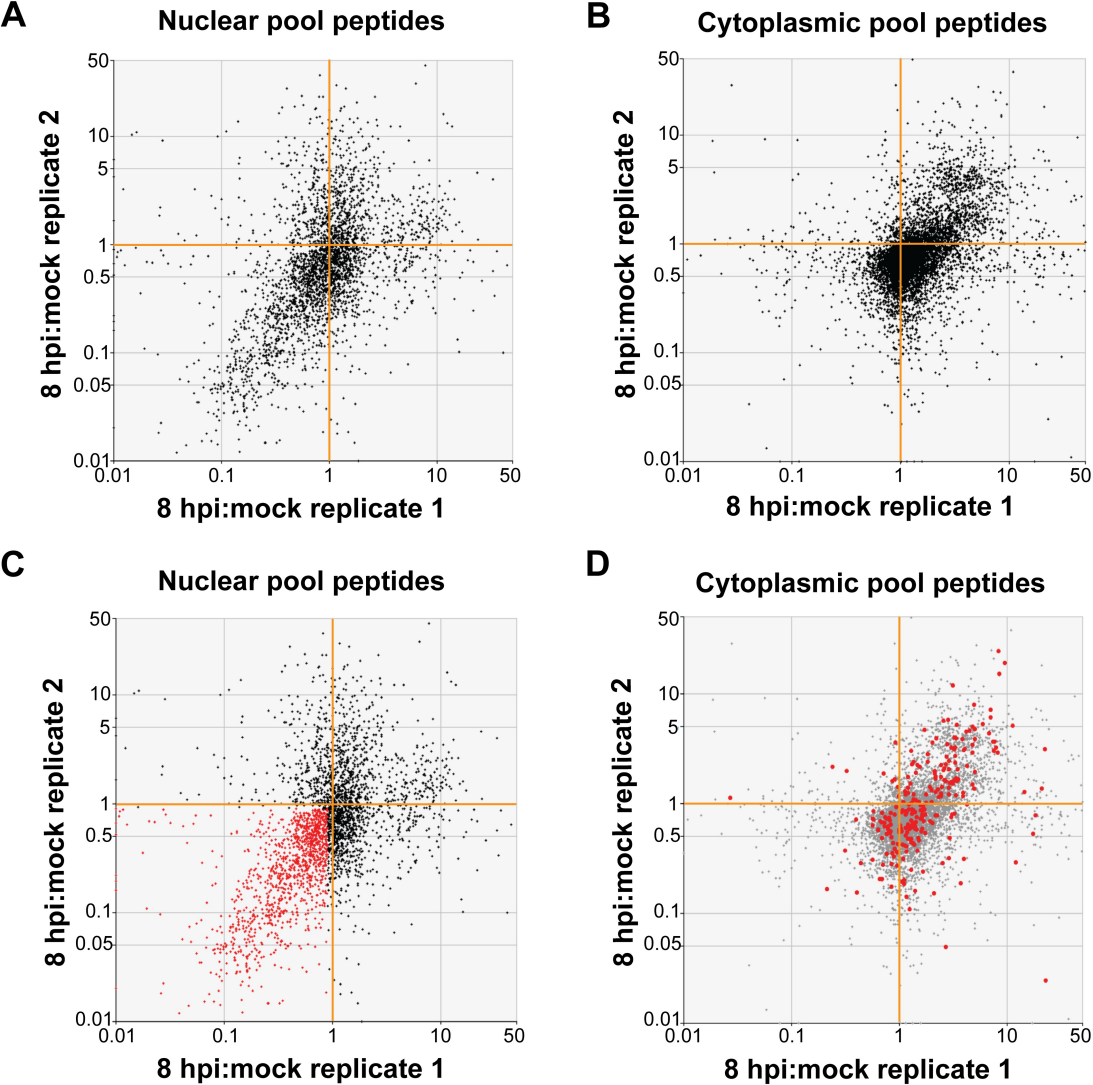
HRV16 induces a coordinated redistribution of proteins into the cytoplasm of infected cells. Scatter plots show 8hpi:mock abundance ratios for all tryptic peptides detected in replicate in (**A**) nuclear and (**B**) cytoplasmic fractions. On the theoretical diagonal line of reproducibility (lower-left to upper-right), the apparent overrepresentation of tryptic peptides in the lower-left quadrant with respect to the upper-right for nuclear tryptic peptides is consistent with a net efflux of proteins from the nucleus, and the corresponding apparent overrepresentation of the upper-right quadrant for cytoplasmic tryptic peptides is consistent with a net influx of proteins into the cytoplasm. (**C**) Tryptic peptides scoring < 0.9, reproducibly, in nuclear 8hpi:mock abundance ratio were recorded (red) and the subset shared with both cytoplasmic replicates was then highlighted (bold red) on the cytoplasmic scatter plot (**D**), indicating a clear coordination between nuclear depletion and cytoplasmic enrichment.

Our second approach to filtering data was at the peptide level. Here, all tryptic peptides detected and quantitated with confidence in replicate samples were subjected to scatter plot analysis (Fig 2). To identify proteins whose nucleocytoplasmic equilibrium was shifted towards the cytoplasm during HRV16 infection, tryptic peptides with 8hpi:mock abundance ratios in nuclear fractions reproducibly 0.9 or less (Fig 2C) were recorded along with their parent accessions then screened, accession-by-accession, for their 8hpi:mock abundance ratios in the cytoplasmic fractions (Fig 2D). A substantial number of accessions with depressed nuclear 8hpi:mock ratios had correspondingly elevated cytoplasmic 8hpi:mock ratios, strongly suggesting a coordinated redistribution of these proteins from nucleus to cytoplasm by 8 hours post-infection with HRV16. It should be noted that this screen could also identify proteins that are blocked from nuclear import following biogenesis in the cytoplasm, due to a breakdown in nucleocytoplasmic transport during infection.

### Validation of mass spectrometry approach

The peptide-level analysis identified factors that have recently been identified as being involved in enterovirus replication (hnRNP M, NONO) but excluded other proteins with known roles in the infectious cycle, such as PCBP2 [45, 46]. One reason for this may have been the presence, in our analysis, of shared peptides with other members of the PCBP family (PCBP1 and PCBP3) which do not re-equilibrate. Another possibility might be that, because PCBP2 is present in the cytoplasm of uninfected cells due to its function as a nuclear shuttling protein, it was unable to pass the thresholds we imposed to identify cytoplasmic enrichment. In our analysis, PCBP2 levels in HRV16-infected HeLa cells did not change significantly in either the cytoplasm or the nucleus with respect to uninfected cells. Our screen was designed to emphasize more dramatic compartmental movements, through changes in normal distribution patterns.

**Fig 3:**
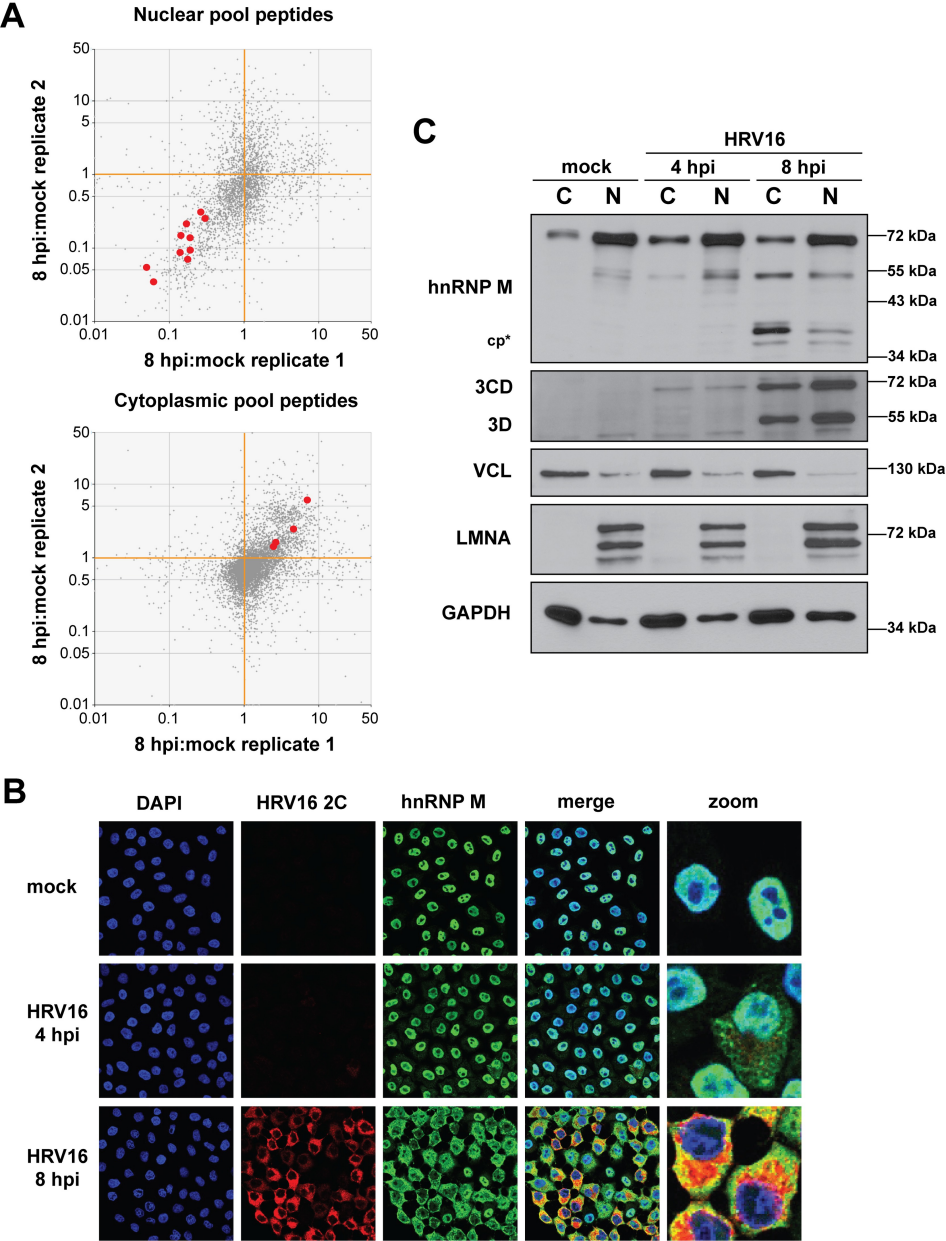
Heterogeneous nuclear ribonucleoprotein M (hnRNP M) redistributes to the cytoplasm of HRV16-infected HeLa cells. (**A**) Tryptic peptides in Fig 2 that correspond to hnRNP M are highlighted red, clearly reflecting the nucleocytoplasmic redistribution of this protein 8 hours post infection (hpi) by HRV16. (**B**) HeLa cells were mock- or HRV16-infected (MOI 10) then fixed 4 or 8 hpi. Cells were permeabilized then probed, via indirect immunofluorescence, for HRV16 2C (red), a marker of HRV16 RNA replication sites, and cellular protein hnRNP M (green). DNA was counterstained with DAPI to indicate location of nuclei (blue). Cells were then imaged using confocal microscopy. (**C**) HeLa cells were mock- or HRV16-infected (MOI 10), fractionated at the indicated times, and fractions were analyzed by Western blot. Cleaved hnRNP M was observed 8 hpi in both the cytoplasmic and nuclear fractions (cp*). HRV16 3D and its precursor 3CD were used as markers of infection. VCL and LMNA were used as markers of the cytoplasmic (C) and nuclear fractions (N), respectively, and GAPDH was used as a general loading control.

To validate the peptide-level analysis and the data more broadly, we selected heterogeneous nuclear ribonucleoprotein M (hnRNP M) as a positive control due to recent work demonstrating the relocalization of this protein to the cytoplasm during poliovirus infection [45]. Peptides corresponding to hnRNP M within our nuclear and cytoplasmic fraction datasets demonstrated clear relocalization by 8 hours post-infection with HRV16 (Fig 3A). We confirmed this redistribution via confocal immunofluorescence microscopy (Fig 3B) and Western blot analysis (Fig 3C). As observed during poliovirus infection, HRV16 infection resulted in the cleavage of hnRNP M, likely as a result of viral 3CD/3C proteinase activity [45]. Additionally, we observed co-localization between HRV16 protein 2C and hnRNP M. This suggested a role for hnRNP M in HRV16 replication, since 2C functions in association with membranous viral RNA replication complexes.

**Fig 4:**
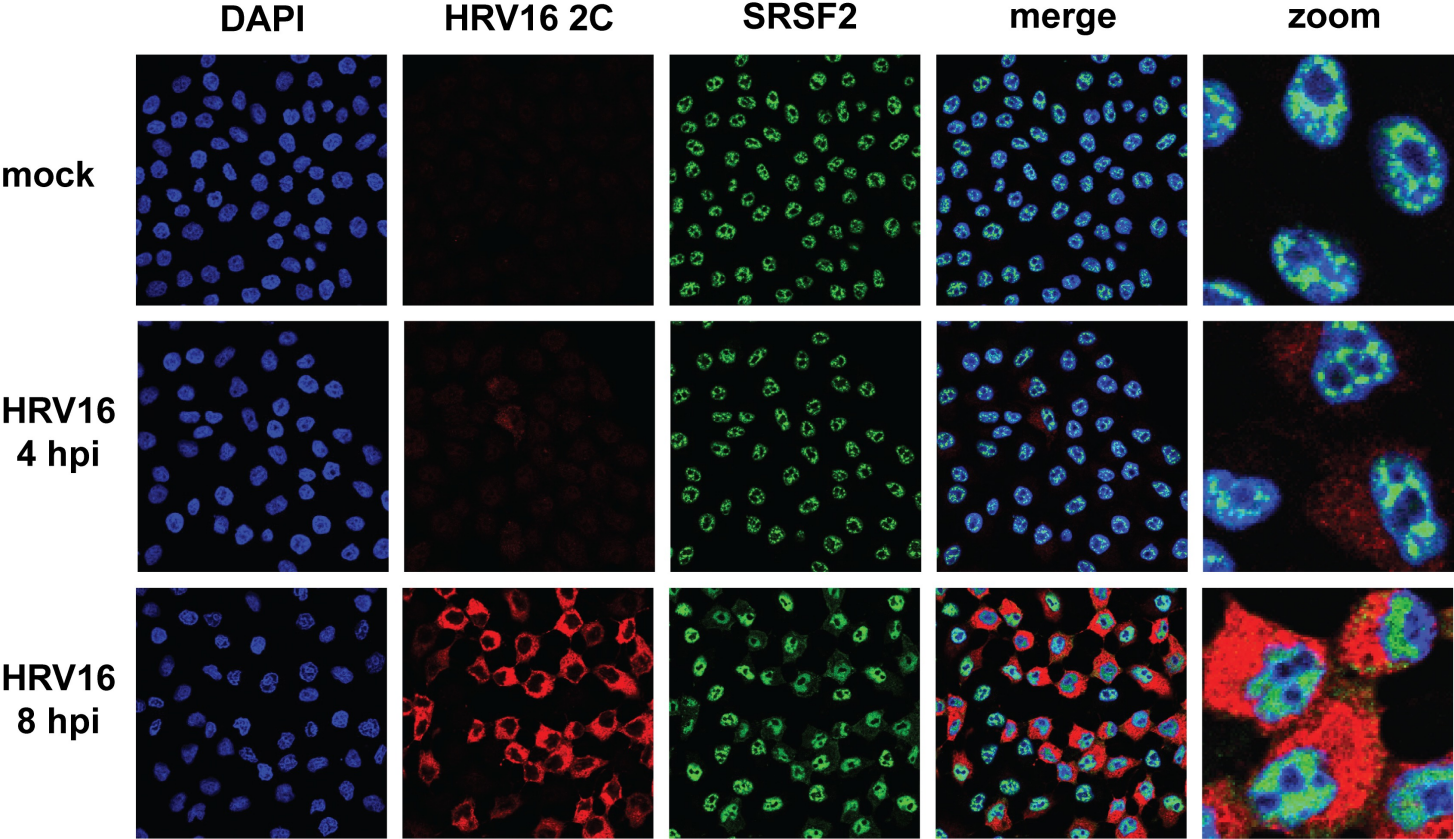
Serine and arginine rich splicing factor 2 (SRSF2) remains within the nucleus of HRV16-infected HeLa cells. HeLa cells were mock- or HRV16-infected (MOI 10) then fixed on coverslips at the indicated times post-infection. HRV16 2C (red) and SRSF2 (green) were labeled by indirect immunofluorescence. DNA was counterstained with DAPI to indicate the location of nuclei (blue). Cells were imaged using confocal microscopy. Hpi: hours post infection.

We selected serine and arginine rich splicing factor 2 (SRSF2, also known as SC35) as a negative control protein, based on previous work with rhinoviruses. Immunofluorescence-based studies have demonstrated that SRSF2 retains its nuclear localization during infection with HRV14 or HRV16 [25, 30]. The distribution of SRSF2 was analyzed via immunofluorescence microscopy, and our results supported the nuclear retention of SRSF2 during HRV16 infection (Fig 4). Taken together, these results highlight the selective redistribution of nuclear proteins during HRV16 infection of HeLa cells.

### Splicing factor proline and glutamine rich (SFPQ) redistributes to the cytoplasm of HRV16-infected HeLa cells

**Fig 5:**
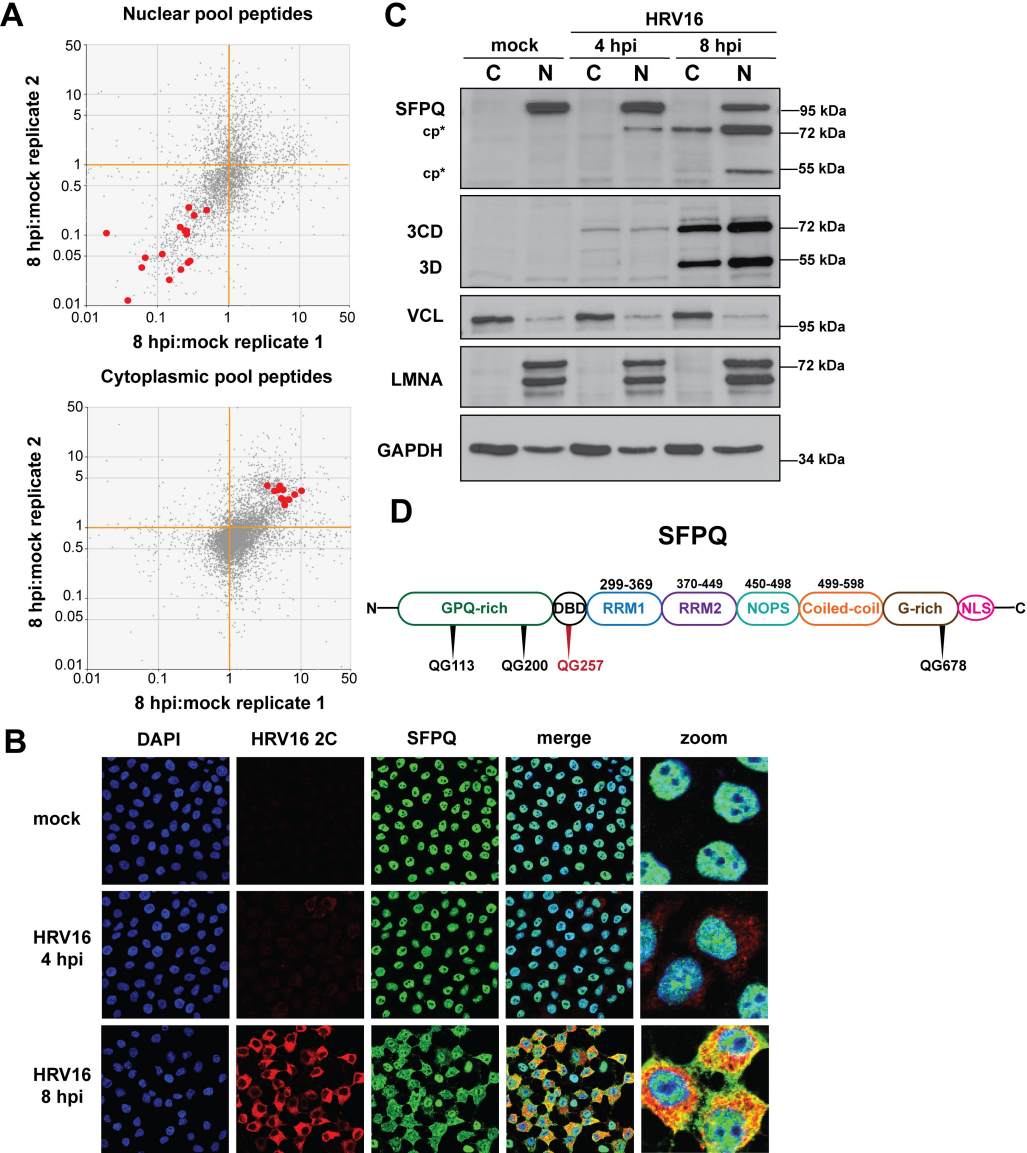
Splicing factor proline and glutamine rich (SFPQ) migrates from the nucleus to the cytoplasm following HRV16 infection. (**A**) Tryptic peptides in Fig 2 that correspond to SFPQ are highlighted red as described in the legend for Fig 3. (**B**) HeLa cells were mock- or HRV16-infected (MOI 10) and fixed then imaged as described in the legend for Fig 4. (**C**) Western blot analysis of cytoplasmic (C) and nuclear (N) fractions of mock- or HRV16-infected HeLa cells. A C-terminal cleavage product (cp*) was detected in the cytoplasm at 8 hours post infection (hpi). A second cleavage fragment of SFPQ was detected in the nucleus at 8 hpi (cp*). HRV16 3D/3CD,VCL, LMNA, and GAPDH used as in Fig 4. (**D**) Schematic of SFPQ domains (adapted from [86]) with putative 3CD/3C cleavage sites. The proposed 3CD/3C cleavage site resulting in the fragment observed 4 hpi is indicated in red. The N-terminal portion of SFPQ includes the glycine, proline, and glutamine-rich (GPQ-rich) and DNA-binding domain (DBD). The C-terminal portion contains two RNA-recognition motifs (RRM1 and RRM2), a NonA/paraspeckle (NOPS) domain, a coiled-coiled domain, glycine-rich (G-rich) region, and nuclear localization signal (NLS).

Based on the compelling evidence for nuclear-cytoplasmic relocalization of splicing factor proline and glutamine-rich (SFPQ) from our protein mass spectrometry data (Fig 5A) and the large number of tryptic peptides identified corresponding to SFPQ in all four mass spectrometry datasets (Table 2), we chose to validate the redistribution pattern of this protein. Immunofluorescence microscopy confirmed the strong nuclear localization of SFPQ in uninfected HeLa cells and its partial redistribution 8 hours post-infection with HRV16, with SFPQ distributed throughout the cell cytoplasm and nucleus at this time (Fig 5B). Fractionation and Western blot analysis of mock- or HRV16-infected HeLa cells revealed that the form of SFPQ that had relocalized to the cytoplasm was a cleavage fragment of the full-length protein (Fig 5C). SFPQ appears as two bands within the nucleus by 4 hours post-infection, only the smaller of which was observed in the cytoplasm at 8 hours post-infection. A second possible cleavage product of SFPQ was detected in the nucleus by 8 hours post-infection. The antibody used in these studies was raised against residues 581–660 of SFPQ, which allowed us to track the C-terminal portion of the 707-amino-acid protein. Due to the apparent cleavage of SFPQ at a time when 3CD proteinase was present in the nucleus, we inspected the amino acid sequence of SFPQ for potential 3CD recognition sites. More than ten such sites were found, including five conforming to the preferred glutamine-glycine (QG) dipeptide. Moreover, utilizing the prediction methods described by Blom and colleagues, which score potential cleavage sites based on sequence and surface exposure, the possible 3CD cleavage sites were narrowed to four QG dipeptides (Fig 5D) [47]. Residue Q257 showed the highest score for predicted cleavage and surface exposure, and cleavage at this site would yield fragments consistent in size with those identified by Western blot analysis. Although SFPQ has an approximate molecular mass of 76 kDa, via SDS-PAGE it has an electrophoretic mobility more consistent with a protein of ~100 kDa. The high probability prediction of SFPQ cleavage by the NetPicoRNA algorithm [47] combined with the observation of C-terminal fragments of SFPQ by Western blot analysis, suggested that SFPQ is targeted for cleavage by viral proteinase 3CD, in the nucleus of HRV16-infected HeLa cells.

### SFPQ is specifically targeted by HRV16 3CD for cleavage

**Fig 6:**
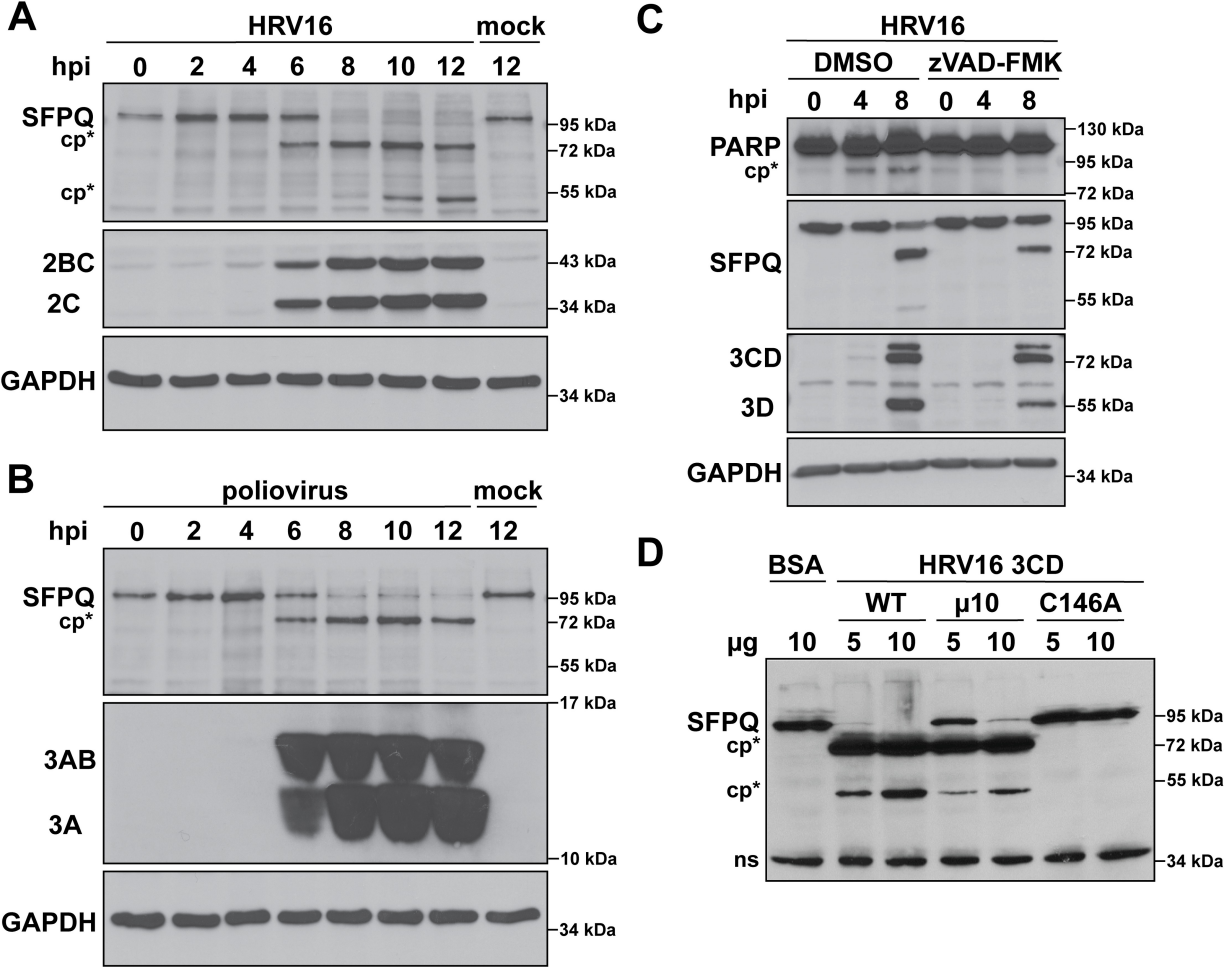
SFPQ is differentially cleaved during HRV16 or poliovirus infection of HeLa cells; cleavage is independent of caspase activity and is carried out by recombinant 3CD *in vitro*. (**A**) HeLa cells were mock- or HRV16-infected (MOI 10), cell lysates were generated at the indicated times, and lysates were subjected to Western blot analysis. SFPQ cleavage products are indicated (cp*). HRV16 2C and its precursor 2BC were used as indicators of infection and GAPDH was used as a loading control. (**B**) HeLa cells were mock- or poliovirus-infected (MOI 10) followed by the generation of cell lysates at the indicated times, and which were then subjected to Western blot analysis. The single SFPQ cleavage product is indicated as in (A). Poliovirus 3A and its precursor 3AB served as markers of infection and GAPDH was used as above. Poliovirus and HRV16 infections were both carried out at 34°C. (**C**) HeLa cells were infected with HRV16 in the presence or absence of 50 μM zVAD-FMK after which lysates were generated at the indicated times and subjected to Western blot analysis. SFPQ cleavage was observed with or without zVAD-FMK. HRV16 3D/3CD and GAPDH were used as above. Cleavage product of poly(ADP-ribose) polymerase 1 (PARP) is indicated (cp*). Data are representative of at least two independent experiments. (D) HeLa cell nuclear extract was incubated with bovine serum albumin (BSA) or different forms of recombinant HRV16 3CD and subjected to Western blot analysis. Cleavage of SFPQ (cp*) was observed in the presence of wild type (WT) 3CD and a form of 3CD containing a mutation in the 3C/3D autoproteolysis site (uncleavable, μ10). Catalytically inactive 3CD (C146A) did not cause cleavage of SFPQ. Hpi: hours post infection; ns: non-specific.

To explore whether SFPQ is a target of 3CD proteinase among the enteroviruses more generally, we tested whether infection with poliovirus resulted in a similar SFPQ cleavage pattern. Western blot analysis of lysates from HRV16- or poliovirus-infected HeLa cells revealed a consistent C-terminal cleavage product (apparent molecular mass of ~72 kDa) produced by either HRV16 or poliovirus at 6 hours post-infection. Poliovirus infection did not, however, lead to detectable amounts of the smaller cleavage product (apparent molecular mass of ~55 kDa) observed at 10 hours post-infection with HRV16 (Fig 6A and 6B). This secondary cleavage product was detected after 8 hours of HRV16 infection in the nucleus of fractionated HeLa cells (Fig 5C). Consistent with their distinct SFPQ cleavage patterns during infection, the 3CD/3C proteinases of HRV16 and poliovirus have distinguishable cleavage site specificities, (reviewed in [48]). If a cellular protease was targeting SFPQ during enterovirus infection, the cleavage pattern for SFPQ would be expected to be identical.

Infection with enteroviruses, specifically poliovirus, has been shown to promote apoptosis, which can result in the degradation of cellular proteins by executioner caspases [49–52]. To determine if SFPQ cleavage resulted from apoptotic induction and subsequent targeting by caspases, HeLa cells were infected with HRV16 in the presence or absence of the cell-permeable pan-caspase inhibitor benzyloxycarbonyl-Val-Ala-Asp-(OMe) fluoromethyl ketone (zVAD-FMK) and lysates generated 0, 4, and 8 hours post-infection. In the absence of zVAD-FMK, a key signature of apoptotic induction was observed by 4 hours post-infection, namely cleavage of the activated caspase substrate poly(ADP-ribose) polymerase 1 (PARP1) [53] (Fig 6C). By contrast, PARP1 remained intact in zVAD-FMK treated cells, indicating that apoptotic cascades were blocked by the inhibitor. In the lysates produced from zVAD-FMK treated cells, the major SFPQ cleavage product (~75 kDa) was detected (Fig 6C), suggesting that SFPQ cleavage was not due to the action of caspases. The overall proportion of cleaved relative to full-length SFPQ was slightly reduced, and the smaller SFPQ fragment absent, in the presence of zVAD-FMK, likely due to the fact that this caspase inhibitor can also suppress viral 2A proteinase activity [54]. Inhibition of 2A proteinase activity could lead to decreased proteolytic processing of the viral polyprotein and reduced levels of mature viral proteins. Caspase-inhibited, HRV16-infected cells resulted in decreased levels of the 3CD proteinase and 3D polymerase, consistent with an inhibition of 2A proteinase activity, and as a result, slight reductions in levels of SFPQ cleavage (Fig 6C). Since SFPQ is cleaved in the presence of a pan-caspase inhibitor and displays a differential cleavage pattern during HRV16 and poliovirus infection, it is likely to be a target of the viral proteinase 3CD/3C during infection.

To demonstrate that SFPQ was being specifically targeted by HRV16 3CD, we performed an *in vitro* cleavage assay with HeLa cell nuclear extract and different forms of recombinant HRV16 3CD (Fig 6D). Incubation with wild type (WT) HRV16 3CD resulted in near complete loss of full-length SFPQ, as did HRV16 3CD containing a mutation adjacent to the 3C-3D cleavage site rendering it uncleavable (μ10), with the higher amount tested [55, 56]. Importantly, the catalytically inactive form of HRV16 3CD (C146A) and the negative control bovine serum albumin (BSA) had no effect on the cleavage state of SFPQ. Furthermore, relative levels of SFPQ cleavage products compared to full-length SFPQ correlated with the amount of 3CD proteinase used in the assay. These results indicate that SFPQ is cleaved by HRV16 3CD during infection of HeLa cells and *in vitro*.

### Knockdown of SFPQ correlates with reduced viral protein production, RNA accumulation and HRV16 titers

**Fig 7:**
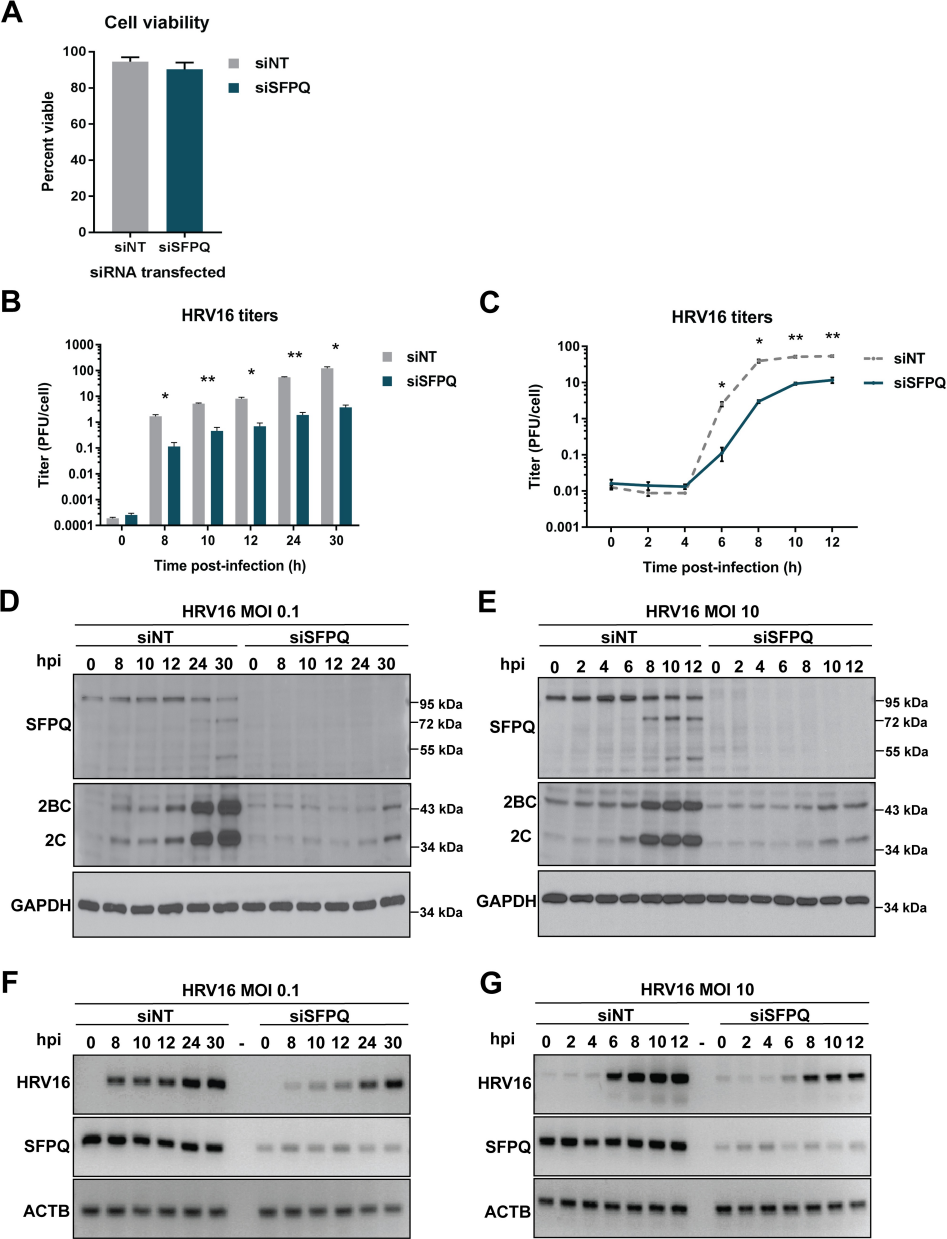
SFPQ knockdown correlates with reduced HRV16 replication. (**A**) Transfection of non-targeting siRNA (siNT) or SFPQ-targeting siRNA (siSFPQ) did not result in statistically significant differences in cell viability 96 h post-transfection, as assessed by trypan blue exclusion prior to infection (*P* > 0.05). Mean percent viable values are displayed with error bars representing one standard deviation. (**B**) HeLa cells transfected with siRNA for 96 h were infected with HRV16 (MOI 0.1) and cells and cell culture fluids were harvested at the indicated times. Virus titer was determined by plaque assay. Data represent the means of three biological replicate experiments with error bars indicating standard error of the means (SEM) (* *P* < 0.005, ** *P* < 0.0005). (**C**) Single cycle growth analysis of HRV16 from siRNA-transfected and HRV16-infected HeLa cells (MOI 10). As in panel B, data represent the means of three biological replicate experiments with error bars displaying SEM (* *P* <0.005, ** *P* <0.0005). (**D**) siRNA-transfected and HRV16-infected HeLa cell lysates corresponding to the time-points in panel B (MOI 0.1) were generated and subjected to Western blot analysis. Knockdown of SFPQ was confirmed and levels of 2C and precursor (2BC) represented viral protein production. GAPDH was used as a loading control. (**E**) Western blot analysis of lysates from siRNA-transfected and HRV16-infected HeLa cells corresponding to time points in panel C (MOI 10). Western blots in panels D and E are representative results from three biological replicate experiments. (**F**) RNA isolated from siRNA-transfected and HRV16-infected HeLa cells at time-points corresponding to panel B (MOI 0.1) was subjected to RT-PCR. Primers for PCR were specific for HRV16 RNA, SFPQ mRNA, or actin (ACTB) mRNA. PCR products were separated by agarose gel electrophoresis. (**G**) RT-PCR analysis of RNA isolated from siRNA-transfected and HRV16-infected HeLa cells at time-points corresponding to panel C (MOI 10). Data in panels F and G are representative results from biological duplicate experiments. hpi: hours post infection.

To determine if SFPQ plays a functional role in the HRV16 infectious cycle, we next explored the effect of siRNA-mediated knockdown of SFPQ on HRV16 replication. Transfection of HeLa cells with an siRNA pool targeting SFPQ (‘siSFPQ’) had no significant effect on cell viability compared to the transfection of a non-targeting siRNA pool (‘siNT’) when viability assays were carried out immediately prior to infection (Fig 7A). Virus titers were then measured to assess the impact of SFPQ knockdown on HRV16 replication (Fig 7B and 7C). Significant reductions in viral titer were observed in SFPQ knockdown cells infected with HRV16 at both low and high multiplicity of infection (MOI). In siSFPQ-transfected cells, virus yield was suppressed ~20-fold between 8 and 30 hours post-infection at low MOI. Single-cycle growth analysis of high MOI infections resulted in similar reductions in virus yield: ~20-fold at 6 and 8 hours post-infection, and ~5-fold at 10 and 12 hours post-infection suggesting a significant delay in the HRV16 replication cycle. Western blot analysis of viral protein production revealed a dramatic reduction in the expression of viral protein 2C and its precursor 2BC in SFPQ knockdown cells (Fig 7D and 7E). The reduction in viral protein expression was consistent with the observed decrease in virus titer. Expression of 2BC/2C at 30 hours after low MOI infection of SFPQ knockdown cells was similar to levels of 2BC/2C protein observed early in infection (8–12 hours post-infection) of control cells, indicating that SFPQ knockdown resulted in a significant delay in viral protein production. Similarly, during high MOI infection of siSFPQ-transfected cells, 2BC/2C production at the end of the infectious cycle (10–12 hours post-infection) was similar to that observed 6 hours post-infection in siNT-transfected cells. SFPQ cleavage was observed to occur concurrently with, or slightly after apparent viral protein production, consistent with our claim that SFPQ is targeted by a viral proteinase (12–24 hours post-infection at low MOI and 6–8 hours post-infection at high MOI). Finally, the effect of SFPQ on viral RNA accumulation was measured by reverse transcription polymerase chain reaction (RT-PCR) of lysates from HRV16-infected, siSFPQ- and siNT-transfected cells. Consistent with viral titers and protein yields, HRV16 RNA accumulation was also reduced and delayed in cells lacking SFPQ (Fig 7F and 7G). Collectively, these results point to a pro-viral role for SFPQ during HRV16 replication in HeLa cells.

### SFPQ is unlikely to play a direct role in viral translation but associates with HRV16 RNA

**Fig 8:**
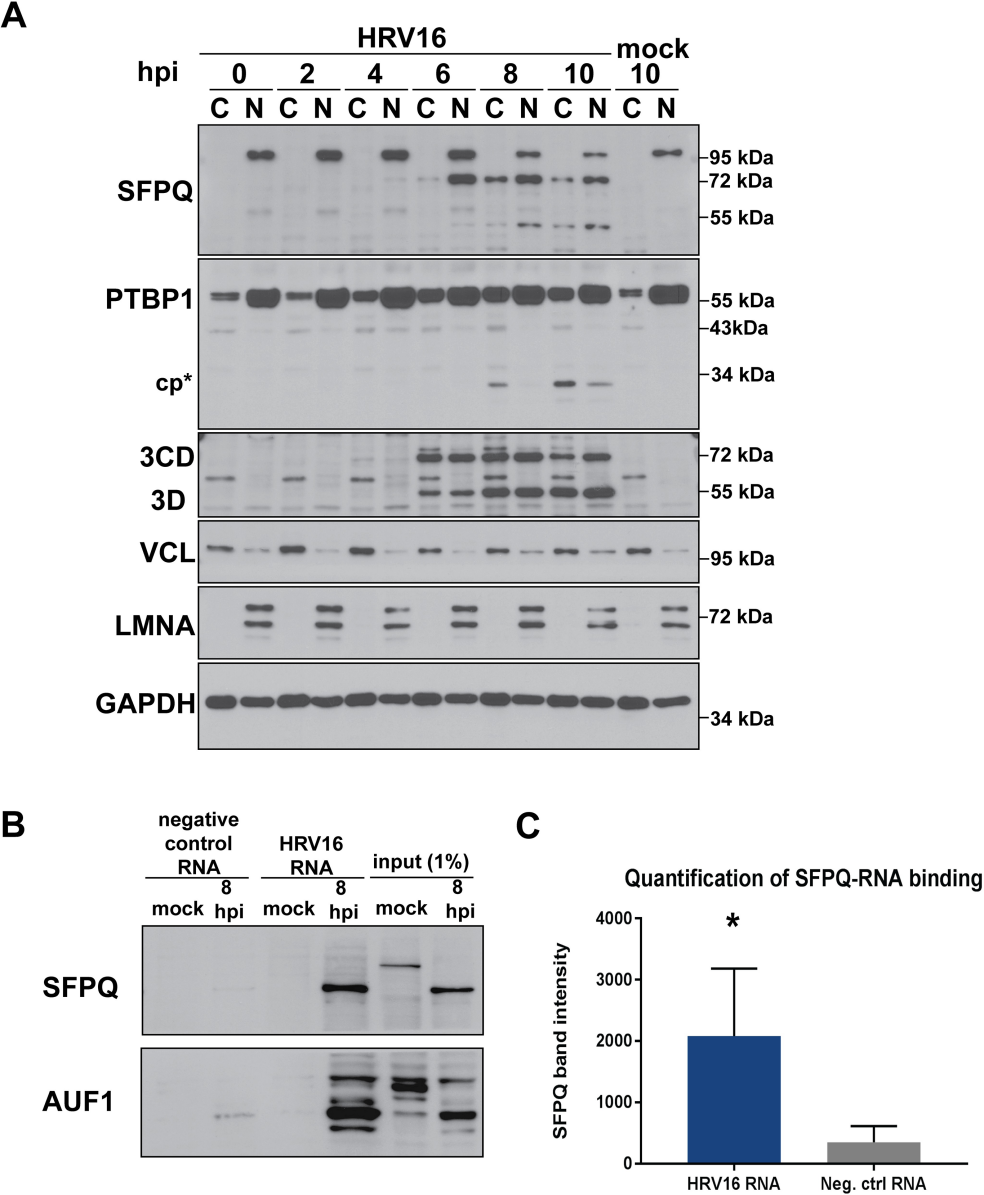
An SFPQ cleavage product associates with *in vitro* transcribed HRV16 RNA. (**A**) HeLa cells were mock- or HRV16 infected, fractionated at 2 h intervals, and the subcellular distribution of proteins was analyzed by Western blot. The cleavage product (cp*) of polypyrimidine tract binding protein 1 (PTBP1) is indicated and HRV16 3D and its precursor 3CD served as markers of infection. VCL and LMNA served as markers for the cytoplasm (C), nucleus (N); GAPDH was used as a general loading control. (**B**) Biotinylated, *in vitro* transcribed control or HRV16 RNA were assayed for binding to cellular proteins present in HeLa cell lysates following mock infection or 8 hours post-infection (hpi) with HRV16 by Western blot analysis. A representative experiment is shown. (**C**) Quantification of four separate RNA affinity experiments was carried out using Quantity One software. Means are shown and error bars represent standard deviations (* *P* < 0.05).

During HRV16 infection, the positive sense RNA genome serves as a template for both translation and genome replication, resulting in the close coupling of these two processes. In an attempt to better understand the role that SFPQ could be playing during the infectious cycle, we compared the timing of SFPQ redistribution with that of the known IRES trans-acting factor (ITAF) PTBP1. As expected, PTBP1 was detected in cytoplasmic extracts of uninfected and infected cells (Fig 8A). This is consistent with its known role in HRV16 IRES-dependent translation, an early step in the replication cycle. Aside from acting as an ITAF, PTBP1 is also involved in mediating the RNA template usage switch that occurs during enterovirus infection, after the accumulation of sufficient levels of viral protein. Because ribosomes and the RNA-dependent RNA polymerase, 3D, travel in opposite directions along the viral mRNA, this template must be cleared of ribosomes prior to the initiation of RNA replication [57–59]. Cleavage of PTBP1 (in combination with cleavage of another ITAF, PCBP2) by the viral 3CD/3C proteinase has been proposed to facilitate this clearing event. The cleaved form of PTBP1, which may be deficient in ribosome recruitment activity, could preclude the binding of intact PTBP1, impeding continued translation [60].

We detected the cleavage of PTBP1 by 8 hours post-infection, at a time when viral protein production is maximal based on the accumulation of 3CD/3D protein. This was consistent with a switch in template usage at later times of HRV16 infection. In direct contrast to PTBP1, the cleaved form of SFPQ was just barely detectable in the cytoplasm at 6 hours post-infection, around the time at which significant levels of viral protein production can be detected. Insofar as SFPQ is not present in the subcellular compartment where viral translation takes place until after protein production has initiated, combined with the fact that the highest levels of cleaved SFPQ are not present in the cytoplasm until 8 hours post-infection, our findings suggest that SFPQ is not required for viral translation to commence.

The presence of SFPQ in the cytoplasm of HRV16-infected HeLa cells at 6 hours post-infection, the time at which RNA replication commences (Fig 7G), together with persistence of the cleavage fragment until at least 10 hours post-infection, would be highly compatible with a role for SFPQ in HRV16 RNA replication. Moreover, the SFPQ C-terminal fragment arising from cleavage at the putative 3CD/3C target site (Q257) would retain both RNA recognition motifs (RRMs) identified within the protein (Fig 5D), suggesting that the C-terminal fragment of SFPQ may retain the RNA-binding properties of the full-length protein. Recently, the RNA sequence UAANGGCU(A/G) was identified as an SFPQ consensus-binding sequence through an approach that combined systematic evolution of ligands by exponential enrichment (SELEX) and cross-linking immunoprecipitation (CLIP) [61]. We note that portions of this consensus sequence can be found within the 5’-NCR as well as the VP4, VP2, 2C, and 3C coding regions of the HRV16 genome [62, 63]. To test whether SFPQ interacts with HRV16 RNA, we assayed the *in vitro* binding of SFPQ from mock- and HRV16-infected HeLa cells to biotinylated, full-length RNA corresponding to the HRV16 genome alongside a negative control RNA. We consistently observed a specific association of the cleaved form of SFPQ present in lysates generated at 8 hours post-infection, with HRV16 RNA (Fig 8, panels B and C). Biotinylated HRV16 RNA was also bound by AU-rich element RNA binding protein 1 (AUF1, also known as hnRNP D) from infected cells, a previously identified HRV16 5’-NCR-binding protein [56]. These results suggested that the C-terminal cleavage product of SFPQ interacts, either directly or via a protein partner, with HRV16 RNA during the mid-to-late stages of infection.

## Discussion

In this study we have applied an unbiased quantitative protein mass spectrometry approach to investigate global changes in protein distribution during HRV16 infection. We detected a coordinated redistribution of proteins, many of which normally function in RNA-related processes such as mRNA splicing, from the nucleus to the cytoplasm by 8 hours post-HRV16 infection of HeLa cells. This suggested that infection with an RNA virus, HRV16, results in the enrichment of a specific functional class of proteins at the site of viral replication. Although a number of the proteins listed in Table 2 may be considered predominantly cytoplasmic, several (e.g., the filamins) also have an important nuclear role that has been recognized more recently [64, 65]. Whether others in our list have nuclear functions that remain to be identified, or whether IRES structures within their mRNAs stimulate their translation during HRV16 infection combined with a virus-induced blockage of nuclear import, remains to be seen.

Although a number of proteins appeared to re-equilibrate to the cytoplasm following infection with HRV16 in HeLa cells, there did appear to be some specificity in this process, insofar as SRSF2, for example, remained within the nucleus throughout infection. We identified the multifunctional nuclear-resident protein SFPQ as a target of the HRV16 3CD/3C proteinase in the nucleus of infected cells and showed that the C-terminal cleavage product moved into the cytoplasm of HRV16-infected cells. Interestingly, SFPQ has been shown to be a transcriptional repressor of the cytokine interleukin-8, and influenza virus or herpes simplex virus infection can localize SFPQ to paraspeckles via interaction with a long noncoding RNA, allowing for transcriptional activation of interleukin-8 [66]. In agreement with this, the distribution of SFPQ within the nucleus 4 hours post-HRV16 infection was observed to condense into distinct puncta, suggesting that SFPQ is relocalized within the nucleus, possibly as part of an innate immune response, before it is targeted by 3DC/3C. However, SFPQ has also been shown to concentrate in nuclear foci upon treatment with actinomycin D, suggesting that the HRV16-induced transcriptional repression could also account for the nuclear puncta observed 4 hours post infection [67].

**Fig 9:**
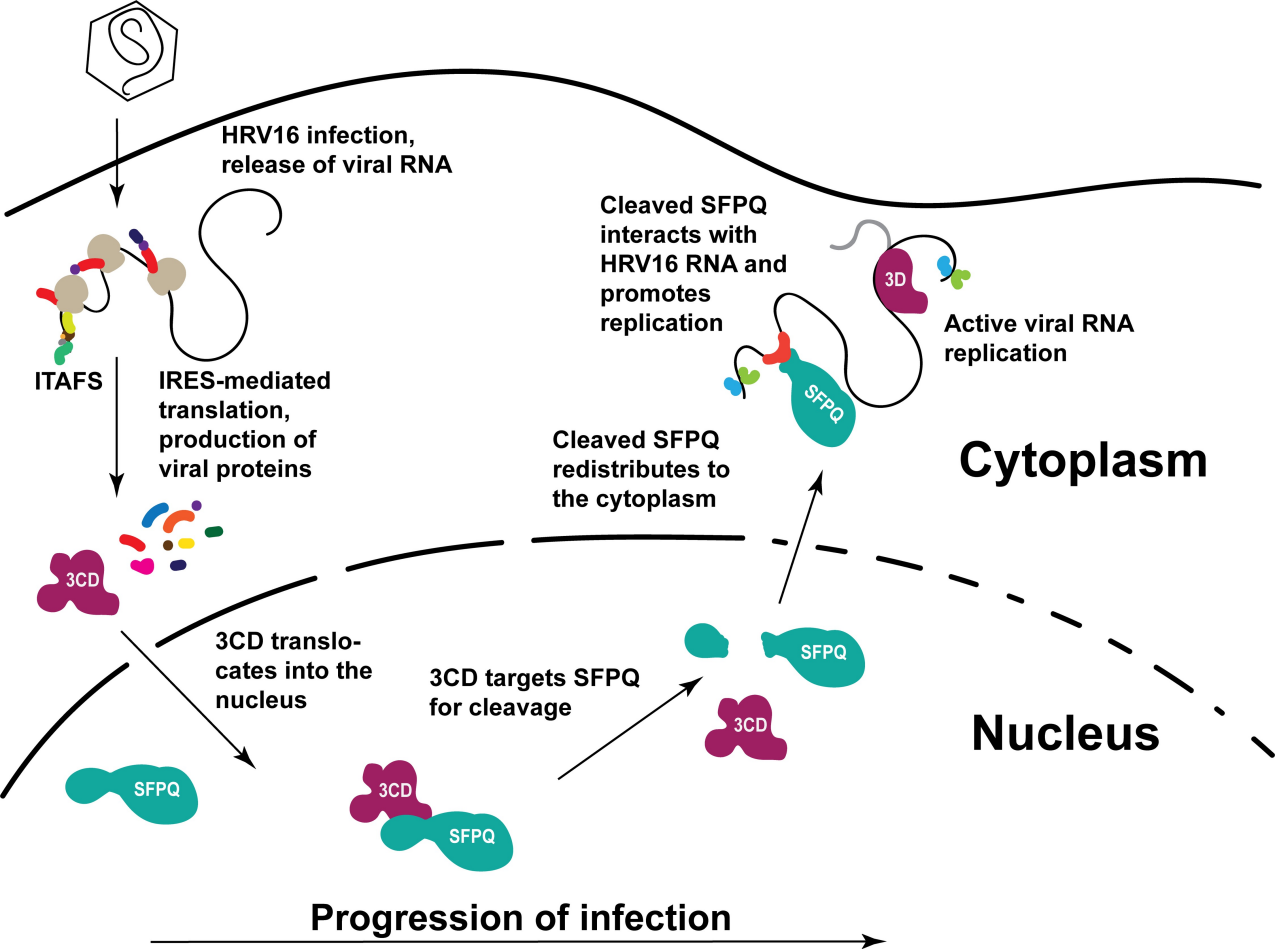
Proposed model of HRV16-SFPQ interactions during the infectious cycle. Following translation of viral proteins, HRV16 3CD/3C enters the nucleus where it targets SFPQ for cleavage at Q257 within the N-terminus. Cleavage releases SFPQ from interactions with nuclear-resident anchors such as DNA and allows the C-terminal fragment to migrate to the cytoplasm through degraded nuclear pore complexes. Once in the cytoplasm, the SFPQ fragment interacts with HRV16 RNA, directly or through other protein partners, and may promote viral replication.

siRNA-mediated knockdown of SFPQ resulted in decreased viral titers, viral protein production, and viral RNA accumulation independent of MOI, suggesting that SFPQ acts as a pro-viral factor. Mechanistically, SFPQ appears to exert its pro-viral function subsequent to viral translation, as it does not relocate from the nucleus until around the time PTBP1 is cleaved and viral RNA synthesis has commenced, about 6–8 hours post-infection. Notably, although only a small proportion of total PTBP1 appeared to be cleaved 6–8 hours-post infection, it is likely that the majority of PTBP1 is not associated with viral replication in infected cells and only the PTBP1 found within HRV16 replication complexes would be targeted for cleavage, allowing for local concentration effects to drive functionality. Finally, we demonstrated an association between the C-terminal cleavage product of SFPQ and *in vitro* transcribed HRV16 RNA, directly or through associated factors, suggesting a direct role for SFPQ in HRV16 replication. Interestingly, we could not detect HRV16 RNA interaction with full-length SFPQ from mock-infected HeLa cells. This suggested that factors associated with SFPQ in uninfected cells preclude HRV16 RNA interaction and/or a viral factor promotes the association of cleaved SFPQ and HRV16 RNA during infection. Through alterations to normal protein distribution patterns, HRV16 is able to increase the functional repertoire of proteins available at the site of replication and to promote viral amplification, including through manipulation of SFPQ. Fig 9 depicts a model of this intracellular scenario.

Aside from the strong evidence for SFPQ redistribution during HRV16 infection (Fig 5 and Table 2), SFPQ is also a compelling candidate for involvement in HRV16 replication due to its association with PTBP1 in uninfected cells [68]. It is possible, for example, that once it has migrated to the cytoplasm of HRV16-infected HeLa cells, the C-terminal fragment of SFPQ sequesters PTBP1 and precludes direct interaction of PTBP1 with HRV16 RNA. In this way, SFPQ could act as a negative regulator in the viral RNA template usage switch from translation to RNA replication that occurs at later times during the infectious cycle. SFPQ has also been reported to be an ITAF of cellular mRNAs. For example, SFPQ can bind and promote IRES-mediated translation of the MYC proto-oncogene, tumor suppressor protein p53, and lymphoid enhancer binding factor 1 (LEF1) [69–71]. Relocalization of SFPQ to the cytoplasm for this purpose was specifically demonstrated for the translation of proteins that are expressed during apoptosis [72]. We show here that the redistribution of SFPQ as a result of HRV16 infection does not occur until after peak times of viral protein production during the infectious cycle (Fig 8A). The fact that knockdown of SFPQ affected both translation and replication of HRV16 was consistent with the close coupling of these two processes: with fewer RNA templates available, less viral protein can be produced. Because siRNA-mediated knockdown of SFPQ suggested a pro-viral role for SFPQ during infection, SFPQ may aid in viral RNA replication (directly or through the recruitment of other proteins), in maintaining the stability of viral RNA, and/or in viral RNA packaging or virion morphogenesis (all of which occur in the later stages of the infectious cycle).

As a first step toward describing the role of SFPQ during HRV16 infection, we demonstrated that a C-terminal cleavage product of this protein produced during infection associated specifically with *in vitro* transcribed, biotinylated HRV16 RNA. This C-terminal fragment is generated in the nucleus of infected cells, where SFPQ is likely a target of the viral 3CD/3C proteinase, and then relocalizes to the cytoplasm. SFPQ cleavage may also preclude it from performing its wide-ranging roles in the nucleus, redirecting the cell to processes favoring viral replication. For example, all arginine-glycine-glycine (RGG) box motifs, present within the GPQ-rich region of SFPQ, and the putative DNA-binding domain, also within the GPQ-rich region, are found in the N-terminal portion of the protein, upstream of the putative 3CD/3C cleavage site at Q257. Cleavage of SFPQ may therefore result in a loss of DNA-binding activity, and the ability of SFPQ to function in transcription and DNA repair may be inhibited.

Rhinoviruses are known to inhibit the nuclear import pathway that utilizes the classical NLS [25], so it is possible that proteolytic separation of the SFPQ C-terminal fragment from nuclear DNA and the retention of newly synthesized SFPQ in the cytoplasm combine to increase the abundance of the SFPQ C-terminal portion in the cytoplasm of HRV16-infected HeLa cells. Importantly, the C-terminal fragment of SFPQ that is present in the cytoplasm of HRV16-infected HeLa cells retains the two RRM domains, presumably allowing SFPQ to bind HRV16 RNA. Aside from the recently identified consensus-binding sequence of SFPQ (UAANGGCU(A/G)), SFPQ has been reported to bind GU-rich sequences, GA-rich sequences, structured RNAs and pyrimidine-rich RNAs, suggesting that SFPQ is polymorphic or promiscuous in its interactions with RNA; the SFPQ binding site(s) within HRV16 RNA remain to be determined [68, 73–77].

Involvement of SFPQ in the replication cycles of other enteroviruses has been suggested by RNA-protein crosslinking studies in which SFPQ was one of several proteins found complexed with poliovirus RNA, another being the non-POU domain containing octamer binding protein (NONO) [46]. No validation experiments or functional characterizations for SFPQ were reported in this study, but NONO-knockdown led to reduced virus yields, and NONO appeared to be a player in poliovirus RNA replication but not IRES-mediated translation. Coxsackievirus B3 (CVB3), another enterovirus, has recently been shown to utilize SFPQ to promote its replication [78]. However, the mechanism proposed for its pro-viral role, namely binding to the 5’-NCR of CVB3 RNA and enhancing IRES-mediated translation, was distinct from that described here for HRV16. The authors suggested that SFPQ relocalizes to the cytoplasm during CVB3 infection via an indirect mechanism involving the phosphorylation of tyrosine 293 (Y293) and dephosphorylation of threonine 687 (T687). Studies of specific tyrosine kinases in cancer cell lines have also linked the phosphorylation of Y293 to a mislocalization of SFPQ to the cytoplasm [79, 80]. Whether phosphorylation of Y293 plays a role during HRV16 infection remains to be seen. During CVB3 infection, SFPQ expression was also demonstrated to be upregulated, possibly via an IRES present within the SFPQ mRNA [78]. However, no cleavage of SFPQ was observed during CVB3 infection, suggesting that although HRV16 and CVB3 both utilize SFPQ for replication, the mechanisms by which it is hijacked are distinct. Interestingly, a Mycobacterium tuberculosis protein has been shown to cleave SFPQ following infection, suggesting convergent evolution in either exploiting its extensive functional capabilities or altering host-cell homeostasis to favor pathogen replication [81]. Additionally, SFPQ has been shown to remain intact during normal cellular apoptosis [82], which lends further credence to the importance of HRV16 3CD/3C in targeting SFPQ specifically.

SFPQ has been shown to be involved in the replication cycles of viruses outside of the *Picornaviridae* family. It mediates the post-transcriptional regulation of HIV-1, both inhibiting the production of HIV-1 transcripts through binding of cis-acting instability elements within gag mRNA and enhancing the production of viral transcripts by binding HIV-1 pre-mRNA [83, 84]. SFPQ is involved in the transcription of influenza A virus RNAs and promotes virus replication [85]. SFPQ also binds the terminal stem-loop regions of hepatitis delta virus RNA of both polarities [74].

SFPQ is a member of the Drosophila behavior human splicing (DBHS) family, which also includes NONO and paraspeckle component 1 (PSPC1). The DBHS proteins are highly multifunctional nuclear factors that are defined through a conserved domain arrangement comprising tandem RNA-recognition motifs (RRM1 and RRM2), a nonA/paraspeckle (NOPS) domain, and a coiled-coiled domain (Fig 5D). The RRM2, NOPS, and coiled-coil domains are required for the formation of homodimers, heterodimers, and extended polymers of the DBHS family members, leading to a molecular scaffold that can mediate diverse cellular functions in pre-mRNA splicing, translation and DNA repair, among others. The ability of these proteins to perform such a variety of functions is likely a result of regulation by post-translational modifications and co-associated factors (reviewed in [86, 87]). Significantly, our cytoplasmic enrichment screen identified all three members of the DBHS protein family (SFPQ, NONO, and PSPC1, Table 2). It is noteworthy that, although SFPQ is considered an essential cellular protein (reviewed in [87]), we have shown here that significant knockdown of SFPQ with a pool of commercially available siRNAs can result in viable HeLa cells up to 96 hours after treatment. It is possible that SFPQ has adequate half-life to fulfill essential functions, or that other DBHS proteins substitute for the loss of SFPQ, or that the absolute requirement for SFPQ is cell-type dependent [88].

In addition to the well-characterized alterations in nucleocytoplasmic trafficking that occur during enterovirus infections, the subcellular location of viral protein 3CD has significant implications for a productive infection. Cellular transcription driven by the three DNA-dependent RNA polymerases (I, II, and III) in mammalian cells is inhibited during infection by enteroviruses. The viral proteinase 3CD/3C inhibits RNA polymerase I-dependent transcription through cleavage of TBP, a factor associated with TATA-box binding protein, RNA polymerase II-dependent transcription through direct cleavage of TBP, and RNA polymerase III-dependent transcription by degradation of general transcription factor IIIC subunit 1 (GTF3C1) [89–93]. The proteinase activity of 3CD/3C also causes cleavage of transcription factors CAMP responsive element binding protein 1 (CREB1), POU class 2 homeobox 1 (POU2F1, also known as Oct-1), and cleavage stimulation factor subunit 2 (CSTF2, also known as CstF-64), with downstream effects on host cell mRNA production and polyadenylation [94–96]. Furthermore, protein 3D of poliovirus has been shown to associate with pre-mRNA processing factor 8 (PRPF8), interfering with splicing and further impacting cellular gene expression [97]. For 3CD and mature 3C and 3D to perform these functions, they must enter the nuclei of infected cells [43]. The data presented here strongly suggest that HRV16 3CD also targets the cellular protein SFPQ for proteolysis in the nucleus.

More than 40 years ago poliovirus replication was shown to be compromised in enucleated cells, and many subsequent studies have reinforced the notion that enteroviruses are dependent upon the nucleus for replication [98]. Nuclear proteins heterogeneous nuclear ribonucleoprotein C (hnRNP C), KH RNA binding domain containing, signal transduction associated 1 (Sam68), hnRNP A1, serine and arginine rich splicing factor 3 (SRSF3/SRp20), and AUF1 have previously been shown to relocalize to the cytoplasm of HRV-infected cells [30, 99–101]. Proteins that have been shown to relocalize during infection with other enteroviruses may do so during HRV16 infection as well. Not all of these proteins necessarily relocalize to the extent observed for SFPQ, as some shuttle between the nucleus and cytoplasm to carry out their normal cellular functions. The multi-functional, DBHS family member SFPQ can now be added to the growing list of nuclear-resident proteins that are involved in human rhinovirus replication resulting, in part, from global changes in host protein distribution and function mediated by the actions of proteinases encoded in the genomes of these cytoplasmic RNA viruses.

## Materials and methods

### Cell culture and virus stocks

HeLa cells (originally obtained from Dr. Eric Stanbridge, University of California, Irvine) were grown as monolayers in Dulbecco’s Modified Eagles Medium (DMEM) supplemented with amphotericin, penicillin, streptomycin, and 8% newborn calf serum (NCS) (complete medium). Cells were maintained in 5% CO2 at 37°C. Virus was generated in HeLa cells transfected with *in vitro* transcribed RNA produced from infectious cDNA clones. Plasmids pRV16.11 (a gift from Dr. Wai-Ming Lee) and pT7PV1 were used to generate HRV16 and Mahoney strain poliovirus, respectively [63, 102]. High titer stocks of virus were generated by serially propagating virus through HeLa cell monolayers (HRV16) or HeLa cells in suspension (poliovirus).

### Virus infections

Stock HRV16 was adsorbed on HeLa cells at the indicated multiplicity of infection (MOI) for 1 h in serum-free DMEM (inoculum) at room temperature. Following adsorption, cells were washed with phosphate buffered saline (PBS). Monolayers were then overlaid with complete medium containing 10 mM MgCl2 and 20 mM HEPES (pH 7.4) (overlay). Immediately following the addition of overlay was regarded as 0 h post-infection. Infected cells were then incubated at 34°C, 5% CO2. Poliovirus infections were performed in the same manner except that adsorption was carried out for 30 minutes. Mock infections were performed simultaneously using serum-free DMEM as inoculum. For caspase inhibition experiments, the HRV16 inoculum and overlay contained 50 μM benzyloxycarbonyl-Val-Ala-Asp-(OMe) fluoromethyl ketone (zVAD-FMK, UBPBio) in DMSO or DMSO alone. At the indicated time-points, cells were washed with PBS and then collected for protein or RNA analysis, or cells and cell culture supernatants were collected for titration by plaque assay.

For plaque assays, cells and culture media were freeze-thawed five times prior to serial dilution in serum-free DMEM and infections were carried out as described above, except that overlay contained 0.45% agarose (Lonza). 72 h following infection a second volume of overlay containing 0.45% agarose was added to cells followed by a further 24 h incubation at 34°C. 96 h after infection, cells were fixed with 10% trichloroacetic acid and then stained with 0.1% crystal violet. Plaques were counted and calculated titers were normalized to the cell count of transfected cells prior to infection (see below). Data are presented as plaque forming units per cell.

### Subcellular fractionation

Cellular fractions of uninfected or infected HeLa cells for mass spectrometry analysis were generated as previously described [42]. Briefly, cell monolayers were washed, scraped, pelleted by centrifugation, and then incubated on ice in reticulocyte standard buffer (RSB; 10 mM NaCl, 10 mM Tris-HCl [pH 7.4], 1.5 mM MgCl_2_) for 5 minutes. Cells were then ruptured by Dounce homogenization. The resulting cell homogenate was subjected to centrifugation (1600 rcf), and the supernatant collected as the cytoplasmic fraction. The pellet was washed with RSB, centrifugation was repeated as above, and this supernatant combined with the cytoplasmic fraction. The remaining pellet (nuclear material) was washed with RSB containing mixed detergent solution (0.4% sodium deoxycholate, 0.9% NP-40) and subjected to centrifugation (1600 rcf). The supernatant was removed and the pelleted nuclei were resuspended in high salt buffer (HSB; 0.5 M NaCl, 50 mM MgCl2, 10 mM Tris-HCl [pH 7.4]) containing DNase I, resulting in the nuclear fraction (crude lysate). In parallel, nucleocytoplasmic fractionation was carried out using NE-PER Nuclear and Cytoplasmic Extraction Reagents (ThermoFisher Scientific) to analyze subcellular distribution of proteins by Western blot.

### Mass spectrometry analysis

#### Tryptic peptides

From each of three cultures (mock-infected, 4 h-infected, 8 h-infected) a total of 9.2x10^7^ HeLa R19 cells were harvested. This number was corrected to 7.5 x10^7^ cells/culture (allowing for ~20% loss during infection and harvesting). From harvested cell pellets, cytoplasmic and nuclear extracts were made in RSB and HSB respectively, as described above. The resulting six fractions from the three cultures provided material for two quantitative mass spectrometry experiments: One employing the three cytoplasmic extracts, the other the three nuclear extracts. Proteins were precipitated from the above extracts by making 8 M in urea/ 0.1 M in triethylammonium bicarbonate (TEAB) then adding 5 volumes of acetone followed by incubation at -20°C for 60 min then centrifugation at 13,000 rpm at 4°C for 60 min. Acetone pellets were each re-dissolved in equivalent volumes of 8 M urea, 0.1 M TEAB, 10 mM Tris(2-carboxyethyl)phosphine (TCEP). Following Bicinchonic acid (BCA) protein assay, an aliquot corresponding to approximately 0.33 mg protein was taken from each of the six samples followed by addition of iodoacetamide to a final concentration of 20–50 mM and 30 min incubation at room temperature in the dark. Dilution to 6 M urea with 0.1 M TEAB was followed by addition of LysC (1:100 trypsin:substrate mass ratio) and incubation at 37°C overnight. After dilution with 0.1 M TEAB to 1 M urea, samples were treated with trypsin (1:50 trypsin:substrate mass ratio) overnight then stimulated for a further 3 h with an equivalent aliquot of trypsin. After mass spectrometric assay for the extent of trypsinization in a small aliquot from each sample, reactions were re-stimulated if necessary with fresh trypsin until fewer than 11% of the identified peptides had missed cleavages. Tryptic digestion products were purified using Sep-Pak C18 (Waters Inc.), eluting with 80% CH_3_CN/0.1% formic acid (FA), then evaporating to dryness under vacuum. After re-dissolving in 0.1 M TEAB, peptides were labeled with isotopically light, intermediate and heavy dimethyl groups [103] for the 4h, 8h and mock cultures, respectively (cytoplasmic) or mock, 4 h and 8 h cultures, respectively (nuclear), followed by quenching with ammonium hydroxide then acidification with FA. The three differentially-labeled samples were combined and the mixture subjected to C18 solid-phase extraction using Sep-Pak C18 as above. Tryptic peptides were re-dissolved in strong cation exchange (SCX) solvent A (30% CH_3_CN, 0.05% (w/v) H_3_PO_4_, KOH to pH 2.7) for loading on a Polysulfoethyl A (200 x 4.6 mm, 5-um particle size, 300 Å pore size) column (PolyLC Inc.) that had been thoroughly pre-equilibrated with solvent A using a Waters 600E multisolvent delivery system/486 detector and monitoring OD_280_ with Clarity chromatography software (DataApex Inc.). After washing with SCX solvent A until the OD_280_ approached zero, the column was eluted with a linear gradient of 6–24% SCX solvent B (solvent A plus 0.5 M KCl) at a flow rate of 0.7 mL/min over 144 min, collecting 1.4 mL fractions. The volume of each fraction was reduced under vacuum to 0.1–0.2 mL prior to C18/SCX stage-tipping [104]. Each stage-tip elution was dried then re-dissolved in 0.1% FA in water for injection, via an Easy-nLC 1000 (ThermoFisher, Inc.), to a 250 x 0.075 mm (ID) nanospray tip packed with ReproSil-Pur C18-AQ (1.9 μm diameter; Dr. Maisch GmbH) pre-equilibrated with 0.1% FA in water. NanoLC-MS/MS. Spectra were acquired using an LTQ Orbitrap Velos Pro (ThermoFisher, Inc.) while running a bipartite linear gradient of 5–23% C18 solvent B (CN3CN in 0.1% FA/water) over 205 min followed by 23–35% C18 solvent B over 30 min at a flow rate of 250 nL/min. In each precursor spectrum (profile; resolution = 60000) the 20 most intense ions above a threshold of 1000 counts with a charge of +2 or greater were subjected to CID activation in the ion trap followed by the generation of a rapid trap-scan centroid fragmentation spectrum. Ions otherwise eligible for fragmentation a second time within a period of 40 sec were added to a 500 member (maximum) exclusion list for a period of 30 sec unless expiring from the list earlier on the basis of either priority or increased signal:noise (S:N) by a factor of 2.0. The above experiment was repeated (in biological replicate), using light, intermediate and heavy dimethyl isotopes for 8 h, mock and 4 h cultures, respectively (both cytoplasmic and nuclear fractions). Data: Target/decoy searches of raw data files were against SwissProt (taxonomy: Human, Rhinovirus Type 16) plus a database of common contaminants with trypsin specificity, allowing 1 missed cleavage, charge state of +2 to +4, Carbamidomethyl (C) as fixed modification and Oxidation(M), deamidated (NQ) as variable modifications, with precursor and product mass tolerances of ±20.00 ppm and ±0.50 Da, respectively. Search results from all fractions of a single SCX gradient (above) were combined, then thresholded to < 5% false discovery rate (FDR), and the identified peptide ions in precursor spectra were quantitated using Mascot Distiller 2.5 with ‘Dimethylation [MD]’ quantitation method allowing N-terminal and lysine labeling with light, medium and heavy reagent. Using in-house software, quant data exports from Mascot Distiller were filtered for good quantitation statistics (> 80% correlation between experimental peak and triple channel peak model, < 40% of total intensity within the triple channel window of a time-integrated ion peak that did not fit the model, < 0.4 in std. error for plots of modeled single channel intensity vs. partner channel intensity for all precursor spectra covering an extracted ion peak).

#### Protein-level analysis

See legend to S2 Table.

#### Peptide-level analysis

From the filtered data, scatter plots were generated of 8hpi:mock isotope intensity ratios for all tryptic peptides common to the duplicate nuclear datasets. Tryptic peptides for which both 8hpi:mock relative quant ratios were < 0.9 (<~50th percentile in the ascending distributions of all ratios) were recorded. After generating a scatter plot from the replicate cytoplasmic data, as above, tryptic peptides were highlighted in the latter plot corresponding to the recorded nuclear tryptic peptides and/or all tryptic peptides from their corresponding accessions.

### Western blot analysis

HeLa whole cell lysates were generated using radioimmunoprecipitation assay (RIPA) buffer (150 mM NaCl, 1% NP-40, 0.5% sodium deoxycholate, 0.1% sodium dodecyl sulfate, 50 mM Tris-HCl [pH 8.0], Pierce Protease Inhibitor Tablet EDTA-free [ThermoFisher Scientific]) after harvesting and pelleting in PBS (4°C, 1200 rcf, 5 minutes). Protein concentration in lysates was determined using the RC DC protein assay (Bio-Rad Laboratories) and equivalent amounts of protein were boiled for 3 minutes in 1x Laemmli sample buffer (LSB) and resolved by 12.5% sodium dodecyl sulfate polyacrylamide gel electrophoresis (SDS-PAGE). Proteins were transferred to an Immobilon-P membrane (Millipore), cut for analysis with multiple primary antibodies simultaneously, and blocked with 5% non-fat milk in phosphate buffered saline containing 0.1% Tween-20 (PBST). Membranes were then incubated for 1 h with primary antibody diluted in PBST containing 5% bovine serum albumin (BSA), washed with PBST, then incubated for 1 h with 1:7500 dilution of goat anti-rabbit or goat anti-mouse horseradish peroxidase (HRP)-conjugated IgG-heavy and light chain secondary antibody (Bethyl Laboratories) diluted in PBST. Membranes were washed with PBST then exposed to Pierce ECL Western Blotting Substrate (ThermoFisher Scientific) and exposed to Blue Autoradiography Film (USA Scientific) and developed. When necessary, membranes were stripped with harsh stripping buffer (2% SDS, 62.5 mM Tris-HCl [pH 6.8], 0.8% 2-mercaptoethanol), washed extensively with water and PBST, then subjected to the same Western blotting procedure described above.

The primary antibodies and corresponding dilutions used were as follows: 1:10,000 vinculin (Abcam ab129002), 1:10,000 GAPDH (Abcam ab181602), 1:2000 lamin-A (Bethyl A303-432A), 1:1000 HRV16 3D [55], 1:750 hnRNP M (Santa Cruz Biotechnology sc20002), 1:750 PSF/SFPQ (Santa Cruz Biotechnology sc374502), 1:15,000 HRV16 2C (antisera provided Dr. Roberto Solari), 1:2000 poliovirus 3A (a gift from Dr. George Belov), 1:1000 PARP (Abcam ab32071), 1:1000 PTBP1 (Abcam ab30317), and 1:10,000 AUF1 (Millipore 07–206).

### Confocal immunofluorescence microscopy

HeLa cells were seeded on glass coverslips and subsequently infected with HRV16 as described above. At the specified time following infection, cells were fixed with 3.7% formaldehyde in PBS for 15 minutes then washed with PBS. Cells were permeabilized with 0.5% NP-40 in PBS for 5 minutes, rinsed with 1% NCS in PBS, then blocked with 5% fetal bovine serum in PBS. Permeabilized cells were then incubated with a mixture of primary antibodies targeting cellular and viral proteins (1:50 hnRNP M [Santa Cruz Biotechnology sc20002], 1:50 PSF/SFPQ [Santa Cruz Biotechnology sc374502], or 1:500 SC35/SRSF2 [Sigma-Aldrich s4045] and 1:2500 HRV16 2C [antisera provided Dr. Roberto Solari]) diluted in PBS containing 5% BSA. Cells were washed with 1% NCS in PBS then incubated with a mixture of secondary antibodies in 1% BSA (1:250 goat anti-rabbit IgG-heavy and light chain DyLight 650-conjugated and 1:250 goat anti-mouse IgG-heavy and light chain DyLight 488-conjugated [Bethyl]). Following washes with 1% NCS in PBS, nuclei were counterstained with 0.4% 4',6-diamidino-2-phenylindole (DAPI), mounted on slides with Fluoro-Gel (Electron Microscopy Sciences) and allowed to dry overnight. Cells were visualized with a laser scanning confocal microscope (Zeiss LSM 700), and images were processed using Zen software (Zeiss).

### *In vitro* cleavage assay

Polyhistidine-tagged wild type HRV16 3CD, uncleavable μ10 3CD (containing a mutation adjacent to the 3C/3D autoproteolysis site), and catalytically inactive C146A 3CD proteins were purified using Ni columns and desalted with PD-10 columns (Amersham). 100 μg HeLa nuclear extract (generously provided by Dr. Klemens Hertel at UC Irvine) was incubated with 5 μg or 10 μg of the respective 3CD protein or 10 μg BSA. Cleavage reactions were carried out in 100 mM KCl, 20 mM HEPES (pH 7.9). Incubation was allowed to proceed for 2 h at 30°C. 30 μl 2x LSB (Laemmli sample buffer) was added to each 50 μl reaction, and 40 μl of each mixture was resolved by SDS-PAGE followed by Western blot analysis.

### siRNA transfections

HeLa cells were transfected using DharmaFECT 1 transfection reagent (Dharmacon T-2001) at 25–33% confluence, 24 h after seeding [105]. Briefly, a pool of siRNAs targeting SFPQ (Dharmacon M-006455-02) or a pool of non-targeting siRNAs (Dharmacon D-001206-13) was incubated with transfection reagent in OPTI-MEM (Gibco) at room temperature for 20 minutes before diluting siRNA to a final concentration of 5 nM in DMEM containing 8% NCS and no antibiotics, then overlaying on PBS-washed HeLa cells. Transfected cells were incubated for 96 h (5% CO2 and 37°C), trypsinized from representative wells, counted using a hemocytometer, and the remaining wells were infected at the indicated MOI as outlined above.

### Reverse transcription polymerase chain reaction

TRIzol (Invitrogen) was added to siRNA-transfected, HRV16-infected cells at the indicated times post-infection, followed by RNA extraction. Complementary DNA (cDNA) was generated from 1 μg of total RNA using either oligo(dT)18 or an HRV16-specific reverse primer listed below and AMV reverse transcriptase (Life Sciences Advanced Technologies). The resulting cDNA was used as a template for polymerase chain reaction (PCR) and indirect analysis of HRV16, SFPQ, and actin beta (ACTB) mRNA levels with 24, 30, or 30 thermal cycler amplification cycles, respectively (Applied Biosystems SimpliAmp). HRV16- or gene-specific primers used were: HRV16-1975-fwd: 5’-CGGGACTGCAAACACTACCT-3’, HRV16-2281-rev: 5’-CCGAAGGCAAAAGTCCTTGC-3’ (58°C annealing temperature [T_a_]), SFPQ-884-fwd: 5’-AGCGATGTCGGTTGTTTGTTG-3’, SFPQ-1096-rev: 5’-AGCGAACTCGAAGCTGTCTAC-3’ (56°C T_a_), ACTBh1-fwd: 5’-CATGTACTGTGCTATCCAGGC-3’, and ACTBh1-rev: 5’-CTCCTTAATGTCACGCACGAT-3’ (56°C T_a_). PCR products were separated by agarose gel electrophoresis and visualized via ethidium bromide staining.

### Affinity pulldown of biotinylated RNA

Detection of proteins interacting with HRV16 RNA was carried out as described previously [106, 107]. Plasmids pRV16.11 and pRstF, a bicistronic luciferase reporter construct, were linearized with EcoICRI or StuI, respectively [108]. DNA was phenol-chloroform extracted and precipitated in ethanol. Linearized templates were transcribed using the MEGAscript T7 Transcription Kit (ThermoFisher Scientific) in the presence of biotin-14-CTP and unlabeled-CTP (1:9) resulting in biotinylated RNA corresponding to the HRV16 genome (~7,200 nt) and a biotinylated negative control RNA (~5,200 nt). Mock- or HRV16-infected HeLa cell lysates were generated with polysome extraction buffer (PEB) (20 mM Tris-HCl [pH 7.5], 100 mM KCl, 5 mM MgCl2, 0.5% NP-40, Pierce Protease Inhibitor Tablet EDTA-free [ThermoFisher Scientific]). 10 μg biotinylated RNA was renatured by incubating in 2x Tris, EDTA, NaCl, Triton (TENT) buffer (20 mM Tris-HCl [pH 8.0], 2 mM EDTA [pH 8.0], 500 mM NaCl, 1% Triton X-100) at 56°C for 5 minutes, 37°C for 5 minutes, then at room temperature for 5 minutes. Renatured, biotinylated RNA was then combined with 500 μg of cell lysate in the presence of ribonuclease inhibitor (Promega Recombinant RNasin) and incubated with intermittent mixing for 1 h. 125 μL of TENT-washed hydrophilic streptavidin magnetic beads (New England Biolabs) were added to mixture and incubation continued for 1 h with intermittent mixing. Beads were washed three times in 1x TENT buffer using a magnetic stand, 40 μL of 1x LSB was added, and beads were heated at 95°C for 5 minutes. The entire sample was then separated by SDS-PAGE followed by Western blot analysis as described above. Quantity One software (Bio-Rad Laboratories) was used to quantify the relative intensity of bands using the volume analysis function.

### Statistical analysis

Statistical analyses employed GraphPad software. All virus titer graphs represent the means of at least 3 biological replicate experiments analyzed in technical triplicate and error is presented as the standard error of the mean (SEM). Cell viability and affinity assay quantification represent the mean and standard deviation (SD) associated with five or four separate assays, respectively. P-values were determined using an unpaired Student’s t test, and statistical significance was established as *P* < 0.05.

## Supporting information

S1 Table Protein mass spectrometry data overview. r1: Total matches, for each of the four datasets, between all MS2 spectra and candidate tryptic peptide sequences in the chosen databases that exceeded the statistical significance threshold for the database search; r2: Initial false-discovery rate (#target vs. #decoy hits at the initial significance threshold); the difference between r3 (accessions) and r4 (‘pFams’, or protein families) reflected the occasional presence of tryptic peptide sequences in multiple members of a pFam); r5 shows the number of successful three-channel quants among all matches on r1 (after ensuring r2 is <5% FDR) in which tryptic peptides occurring within multiple accessions appear multiple times in the (redundant) r5 listing but not in the r1 listing, and in which individual quant channels that had triggered MS2 multiple times were collapsed to a single quant; r6 corresponds to r5 after rendering non-redundant for tryptic peptides shared between accessions (typically within pFams); the total number of peptide sequences (r7) is lower because individual peptide ions were often sequenced in consecutive scans which collapsed into the quant of a single LC peak, and individual tryptic peptide sequences appeared multiple times in multiple modiforms and charge states; comparing r8 to r7 indicates the peak resolution of SCX chromatography (the proportion of peptide sequences appearing in just one SCX fraction); r9 redundantizes r8 by multiply-listing shared tryptic peptides against all accessions in which they occur; r10 –r12 shows the progressive filtering of the set on r9 for quality of quantitation, with a final de-redundantization on r12. The asterisks (*) indicate that p = 0.05 yielded an initial FDR > than our 5% FDR threshold for the project as a whole. For these two samples, the complete list of identified proteins/peptides was re-thresholded with a more stringent p value, to yield an FDR in the range 4.98%–5%, prior to any subsequent steps (including quantitation).
(DOCX)

S2 Table All proteins, from the analysis summarized in S1 Table, whose abundance increased in the cytoplasm while decreasing in the nucleus at 8 hr post-infection of HeLa cells with HRV16. Values under each of the four dataset columns (‘Nuc1’, ‘Nuc2’, ‘Cyto1’, ‘Cyto2’) take the form ‘x/y/z’ in which an 8hr:mock abundance ratio of x (geometric mean of relevant, quantifiable tryptic peptides) was based on a total of z tryptic peptide species, y of which tracked the direction (< 1 or > 1) of x. Re-equilibration could result from virus-induced efflux from the nucleus and/or inhibition of nuclear import. See text for details.
(DOCX)
