# Supplementary material for: Exploitation of nuclear functions by human rhinovirus, a cytoplasmic RNA virus
Source: PLoS Pathog. 2018 Aug 24;14(8):e1007277. doi: 10.1371/journal.ppat.1007277 (PMC6126879; doi:10.1371/journal.ppat.1007277)
Supplement: S1 Table — r1: Total matches, for each of the four datasets, between all MS2 spectra and candidate tryptic peptide sequences in the chosen databases that exceeded the statistical significance threshold for the database search; r2: Initial false-discovery rate (#target vs. #decoy hits at the initial significance threshold); the difference between r3 (accessions) and r4 (‘pFams’, or protein families) reflected the occasional presence of tryptic peptide sequences in multiple members of a pFam); r5 shows the number of successful three-channel quants among all matches on r1 (after ensuring r2 is <5% FDR) in which tryptic peptides occurring within multiple accessions appear multiple times in the (redundant) r5 listing but not in the r1 listing, and in which individual quant channels that had triggered MS2 multiple times were collapsed to a single quant; r6 corresponds to r5 after rendering non-redundant for tryptic peptides shared between accessions (typically within pFams); the total number of peptide sequences (r7) is lower because individual peptide ions were often sequenced in consecutive scans which collapsed into the quant of a single LC peak, and individual tryptic peptide sequences appeared multiple times in multiple modiforms and charge states; comparing r8 to r7 indicates the peak resolution of SCX chromatography (the proportion of peptide sequences appearing in just one SCX fraction); r9 redundantizes r8 by multiply-listing shared tryptic peptides against all accessions in which they occur; r10 –r12 shows the progressive filtering of the set on r9 for quality of quantitation, with a final de-redundantization on r12. The asterisks (*) indicate that p = 0.05 yielded an initial FDR > than our 5% FDR threshold for the project as a whole. For these two samples, the complete list of identified proteins/peptides was re-thresholded with a more stringent p value, to yield an FDR in the range 4.98%–5%, prior to any subsequent steps (including quantitation). (DOCX) [file ppat.1007277.s001.docx]

**Supplementary Table S1**. **Protein mass spectrometry data overview.** r1: Total matches, for each of the four datasets, between all MS2 spectra and candidate tryptic peptide sequences in the chosen databases that exceeded the statistical significance threshold for the database search; r2: Initial false-discovery rate (#target vs. #decoy hits at the initial significance threshold); the difference between r3 (accessions) and r4 (‘pFams’, or protein families) reflected the occasional presence of tryptic peptide sequences in multiple members of a pFam); r5 shows the number of successful three-channel quants among all matches on r1 (after ensuring r2 is <5% FDR) in which tryptic peptides occurring within multiple accessions appear multiple times in the (redundant) r5 listing but not in the r1 listing, and in which individual quant channels that had triggered MS2 multiple times were collapsed to a single quant; r6 corresponds to r5 after rendering non-redundant for tryptic peptides shared between accessions (typically within pFams); the total number of peptide sequences (r7) is lower because individual peptide ions were often sequenced in consecutive scans which collapsed into the quant of a single LC peak, and individual tryptic peptide sequences appeared multiple times in multiple modiforms and charge states; comparing r8 to r7 indicates the peak resolution of SCX chromatography (the proportion of peptide sequences appearing in just one SCX fraction); r9 redundantizes r8 by multiply-listing shared tryptic peptides against all accessions in which they occur; r10 – r12 shows the progressive filtering of the set on r9 for quality of quantitation, with a final de-redundantization on r12. The asterisks (*) indicate that p = 0.05 yielded an initial FDR > than our 5% FDR threshold for the project as a whole. For these two samples, the complete list of identified proteins/peptides was re-thresholded with a more stringent p value, to yield an FDR in the range 4.98% - 5%, prior to any subsequent steps (including quantitation).

| **Row** |  | **Nuclear1** | **Nuclear2** | **Cyto1** | **Cyto2** |
| --- | --- | --- | --- | --- | --- |
|  | SCX fractions analyzed: | 36 | 36 | 39 | 36 |
|  | **Database search result** |  |  |  |  |
| r1 | Total peptide-spectrum matches (non-redundant): | 123,946 | 40,676 | 54,023 | 113,488 |
| r2 | Initial FDR (homology): | 2.99% | 9.03%* | 6.48%* | 3.94% |
|  | **Quant export** |  |  |  |  |
| r3 | Total unique accessions: | 5,311 | 4,617 | 4,929 | 6,929 |
| r4 | Total unique pFams: | 4,972 | 4,336 | 4,661 | 6,318 |
| r5 | Total peptide 3-channel triplets (redundant, for accessions): | 136,543 | 43,014 | 61,236 | 127,575 |
|  | **In house counts** |  |  |  |  |
| r6 | Non-redundant (for protein homologs) peptide 3-channel triplets: | 61,298 | 23,003 | 33,770 | 59,209 |
| r7 | Unique peptides sequences: | 21,650 | 14,284 | 14,844 | 36,545 |
| r8 | Σ (unique peptides sequences per SCX fraction): | 27,377 | 14,284 | 17,851 | 36,545 |
| r9 | Redundant (for protein homologs) r8: | 41,164 | 23,600 | 23,491 | 69,783 |
|  | **In house filtering** |  |  |  |  |
| r10 | Non-keratin r9: | 39,432 | 23,043 | 23,063 | 68,758 |
| r11 | r10 passing all quant quality filters: | 24,037 | 5,750 | 10,849 | 24,727 |
| r12 | Non-redundant r11: | 18,864 | 4,651 | 9,011 | 19,295 |
|  | **In house scatter** |  |  |  |  |
|  | Shared nuclear (r12): | 1,631 | |  |  |
|  | Shared cyto (r12): |  |  | 4,110 | |
