# Supplementary material for: Exploitation of nuclear functions by human rhinovirus, a cytoplasmic RNA virus
Source: PLoS Pathog. 2018 Aug 24;14(8):e1007277. doi: 10.1371/journal.ppat.1007277 (PMC6126879; doi:10.1371/journal.ppat.1007277)
Supplement: S2 Table — Values under each of the four dataset columns (‘Nuc1’, ‘Nuc2’, ‘Cyto1’, ‘Cyto2’) take the form ‘x/y/z’ in which an 8hr:mock abundance ratio of x (geometric mean of relevant, quantifiable tryptic peptides) was based on a total of z tryptic peptide species, y of which tracked the direction (< 1 or > 1) of x. Re-equilibration could result from virus-induced efflux from the nucleus and/or inhibition of nuclear import. See text for details. (DOCX) [file ppat.1007277.s002.docx]

**Supplementary Table S2.** **All proteins, from the analysis summarized in Table S1, whose abundance increased in the cytoplasm while decreasing in the nucleus at 8 hr post-infection of HeLa cells with HRV16.** Values under each of the four dataset columns (‘Nuc1’, ‘Nuc2’, ‘Cyto1’, ‘Cyto2’) take the form ‘x/y/z’ in which an 8hr:mock abundance ratio of x (geometric mean of relevant, quantifiable tryptic peptides) was based on a total of z tryptic peptide species, y of which tracked the direction (< 1 or > 1) of x. Re-equilibration could result from virus-induced efflux from the nucleus and/or inhibition of nuclear import. See text for details.

| **Accession** | **Description** | **Nuc1** | **Nuc2** | **Cyto1** | **Cyto2** |
| --- | --- | --- | --- | --- | --- |
| 6PGL_HUMAN | 6-phosphogluconolactonase | 0.4147/3/3 |  | 1.0082/5/7 |  |
| ABLM1_HUMAN | Actin-binding LIM protein 1 | 0.1907/7/7 |  |  | 1.266/1/1 |
| ACINU_HUMAN | Apoptotic chromatin condensation inducer in the nucleus |  | 0.3979/8/8 |  | 3.0097/4/4 |
| ACSL4_HUMAN | Long-chain-fatty-acid--CoA ligase 4 | 0.0322/2/3 | 0/1/1 | 2.0131/4/4 |  |
| ACTBL_HUMAN | Beta-actin-like protein 2 | 0.1351/10/11 |  |  | 2.189/4/9 |
| ACTC_HUMAN | Actin, alpha cardiac muscle 1 | 0.137/18/19 |  |  | 2.4417/2/9 |
| ADAS_HUMAN | Alkyldihydroxyacetonephosphate synthase, peroxisomal | 0.3047/1/2 |  | 6.434/1/1 | 2.0968/10/11 |
| AL3B1_HUMAN | Aldehyde dehydrogenase family 3 member B1 | 0.4968/1/1 |  | 12.7593/2/2 | 1.2133/3/3 |
| AL9A1_HUMAN | 4-trimethylaminobutyraldehyde dehydrogenase | 0.0152/1/1 |  | 1.9628/4/4 |  |
| ANX11_HUMAN | Annexin A11 | 0.473/1/1 |  | 2.4844/4/4 | 1.173/4/7 |
| ANXA2_HUMAN | Annexin A2 | 0.4317/21/23 | 0.1666/8/8 | 2.0875/14/16 | 1.2723/18/18 |
| ANXA4_HUMAN | Annexin A4 | 0.578/1/1 |  | 2.5252/2/2 | 1.5649/6/6 |
| ANXA6_HUMAN | Annexin A6 | 0.132/7/7 | 0.0816/6/6 | 2.7491/6/6 | 1.3958/7/7 |
| ARI4B_HUMAN | AT-rich interactive domain-containing protein 4B | 0.1797/2/2 |  |  | 133.3/1/1 |
| ARPC4_HUMAN | Actin-related protein 2/3 complex subunit 4 | 0.3164/2/2 | 0.298/2/2 | 4.0387/3/3 |  |
| ARRB1_HUMAN | Beta-arrestin-1 | 0.3935/1/1 |  |  | 1.2/1/1 |
| ASCC3_HUMAN | Activating signal cointegrator 1 complex subunit 3 |  | 0.0002/1/1 |  | 4.7205/1/2 |
| AT2C1_HUMAN | Calcium-transporting ATPase type 2C member 1 | 0.3569/1/1 |  |  | 1.629/1/1 |
| ATAD2_HUMAN | ATPase family AAA domain-containing protein 2 | 0.7275/5/6 | 0.3777/1/1 |  | 8.775/1/1 |
| ATX10_HUMAN | Ataxin-10 | 0.6603/2/2 |  | 1.0063/1/5 |  |
| BCORL_HUMAN | BCL-6 corepressor-like protein 1 | 0.4972/2/2 |  |  | 3.266/1/1 |
| CALD1_HUMAN | Caldesmon | 0.2418/2/2 | 0.491/1/1 |  | 1.4977/2/2 |
| CATC_HUMAN | Dipeptidyl peptidase 1 | 0.0343/1/2 |  | 2.329/1/1 |  |
| CAV1_HUMAN | Caveolin-1 | 0.5884/2/2 |  |  | 3.225/1/1 |
| CBS_HUMAN | Cystathionine beta-synthase | 0.5551/2/2 |  | 1.0214/3/5 |  |
| CCAR1_HUMAN | Cell division cycle and apoptosis regulator protein 1 |  | 0.5382/3/3 |  | 1.5844/4/4 |
| CD44_HUMAN | CD44 antigen | 0.6967/1/4 | 0.2408/1/1 | 2.2396/4/4 | 1.269/4/5 |
| CENPF_HUMAN | Centromere protein F |  | 0.0541/4/5 |  | 2.096/1/1 |
| CF120_HUMAN | UPF0669 protein C6orf120 | 0.1204/1/1 |  |  | 2.1289/1/1 |
| CHD4_HUMAN | Chromodomain-helicase-DNA-binding protein 4 | 0.7135/29/29 |  |  | 1.8152/3/3 |
| CHSTE_HUMAN | Carbohydrate sulfotransferase 14 | 0.0001/1/1 |  |  | 110.7/1/1 |
| CK5P2_HUMAN | CDK5 regulatory subunit-associated protein 2 |  | 0.0012/1/1 |  | 178.4/1/1 |
| CK5P3_HUMAN | CDK5 regulatory subunit-associated protein 3 | 0.4037/1/3 |  |  | 1.236/4/4 |
| CMTR1_HUMAN | Cap-specific mRNA (nucleoside-2'-O-)-methyltransferase 1 | 0.7009/2/2 |  | 4.002/1/1 |  |
| COBL_HUMAN | Protein cordon-bleu | 0.3/1/1 | 0.1735/1/1 |  | 1.5899/2/2 |
| COBL1_HUMAN | Cordon-bleu protein-like 1 | 0.7396/1/1 |  |  | 1.3172/1/1 |
| COR1B_HUMAN | Coronin-1B | 0.2592/5/5 | 0.0277/1/1 | 2.2832/3/3 | 2.1327/3/3 |
| COR1C_HUMAN | Coronin-1C | 0.1418/7/7 | 0.0432/3/3 | 25.9097/5/5 | 1.335/6/6 |
| COX41_HUMAN | Cytochrome c oxidase subunit 4 isoform 1, mitochondrial | 0.6916/2/4 |  |  | 3.426/1/1 |
| CP250_HUMAN | Centrosome-associated protein CEP250 |  | 0.0025/1/1 |  | 1.3936/1/3 |
| CP2S1_HUMAN | Cytochrome P450 2S1 | 0.0002/1/1 |  |  | 1.551/1/1 |
| CP51A_HUMAN | Lanosterol 14-alpha demethylase | 0.1113/1/1 |  |  | 1.285/4/4 |
| CPNE3_HUMAN | Copine-3 | 0.4697/2/2 |  | 2.284/3/3 |  |
| CPSF1_HUMAN | Cleavage and polyadenylation specificity factor subunit 1 | 0.2945/10/10 | 0.1265/4/4 |  | 3.0957/2/2 |
| CPSF2_HUMAN | Cleavage and polyadenylation specificity factor subunit 2 | 0.2228/4/5 | 0.1982/2/2 | 2.005/1/1 | 4.5298/6/6 |
| CPSF3_HUMAN | Cleavage and polyadenylation specificity factor subunit 3 | 0.3349/3/3 |  |  | 2.292/1/1 |
| CRIP2_HUMAN | Cysteine-rich protein 2 | 0.342/2/2 |  |  | 1.1927/2/2 |
| CRNL1_HUMAN | Crooked neck-like protein 1 | 0.5663/5/5 | 0.0714/1/1 | 11.3733/1/1 |  |
| CSK_HUMAN | Tyrosine-protein kinase CSK | 0.4442/1/1 |  | 11.4686/2/2 |  |
| CSRP1_HUMAN | Cysteine and glycine-rich protein 1 | 0.1871/2/2 |  | 3.4413/5/5 | 1.6145/1/1 |
| CSTF1_HUMAN | Cleavage stimulation factor subunit 1 | 0.2928/5/5 |  | 6.4939/3/3 | 2.0937/2/2 |
| CSTF3_HUMAN | Cleavage stimulation factor subunit 3 | 0.5483/5/5 | 0.3508/2/2 | 10.25/1/1 | 7.0256/4/4 |
| CTG1B_HUMAN | Cancer/testis antigen 1 | 0.0017/1/1 |  |  | 61.86/1/1 |
| CYTSB_HUMAN | Cytospin-B | 0.1754/2/2 |  |  | 1.3204/2/2 |
| DAZP1_HUMAN | DAZ-associated protein 1 | 0.3577/2/2 |  |  | 1.1738/2/3 |
| DBLOH_HUMAN | Diablo homolog, mitochondrial | 0.2402/1/1 |  |  | 1.364/3/3 |
| DC1L2_HUMAN | Cytoplasmic dynein 1 light intermediate chain 2 | 0.0053/1/1 |  | 2379.5397/1/1 |  |
| DDX17_HUMAN | Probable ATP-dependent RNA helicase DDX17 | 0.3948/22/22 | 0.3764/5/6 | 1.9736/8/8 |  |
| DESM_HUMAN | Desmin | 0.3358/1/6 |  | 2.5665/4/4 | 25.9409/2/2 |
| DHX9_HUMAN | ATP-dependent RNA helicase A | 0.2869/32/34 | 0.2458/8/9 | 2.2561/8/9 | 1.6309/18/18 |
| DIDO1_HUMAN | Death-inducer obliterator 1 |  | 0.5664/4/4 |  | 1.4009/3/3 |
| DJB11_HUMAN | DnaJ homolog subfamily B member 11 | 0.4968/3/6 |  |  | 7.8136/3/3 |
| DREB_HUMAN | Drebrin | 0.2133/9/9 | 0.1965/3/3 | 3.1428/3/3 | 1.2084/4/4 |
| DSC2_HUMAN | Desmocollin-2 | 0.476/1/1 |  |  | 1.385/1/1 |
| DX39A_HUMAN | ATP-dependent RNA helicase DDX39A | 0.5005/5/10 |  | 13.5955/6/6 |  |
| DX39B_HUMAN | Spliceosome RNA helicase DDX39B | 0.6418/6/12 |  | 8.5124/7/7 |  |
| DZIP1_HUMAN | Zinc finger protein DZIP1 | 0.0205/1/1 |  | 18.23/1/1 |  |
| ELAV1_HUMAN | ELAV-like protein 1 | 0.2898/14/14 | 0.3304/1/1 |  | 1.377/3/4 |
| ELAV2_HUMAN | ELAV-like protein 2 | 0.1041/3/4 |  |  | 1.295/1/1 |
| FAF2_HUMAN | FAS-associated factor 2 | 0.0444/1/2 |  | 447.1/1/1 | 2.2276/5/5 |
| FIP1_HUMAN | Pre-mRNA 3'-end-processing factor FIP1 | 0.2878/11/11 | 0.121/1/1 |  | 3.7514/4/4 |
| FLNA_HUMAN | Filamin-A | 0.2329/56/59 | 0.044/22/22 | 3.6386/50/50 | 1.7896/50/52 |
| FLNB_HUMAN | Filamin-B | 0.266/36/37 | 0.0552/7/7 | 2.113/27/27 | 1.2044/38/43 |
| FLOT1_HUMAN | Flotillin-1 | 0.4573/6/6 | 0.1824/1/1 |  | 1.4146/2/2 |
| FLOT2_HUMAN | Flotillin-2 |  | 0.161/1/1 | 2.8295/2/2 |  |
| GLYM_HUMAN | Serine hydroxymethyltransferase, mitochondrial | 0.524/2/13 |  | 2.9321/4/5 | 3.0969/10/11 |
| GPTC4_HUMAN | G patch domain-containing protein 4 |  | 0.5773/3/3 | 2.7978/1/1 |  |
| H2AY_HUMAN | Core histone macro-H2A.1 |  | 0.3803/6/9 |  | 2.9678/2/2 |
| HACD3_HUMAN | Very-long-chain (3R)-3-hydroxyacyl-CoA dehydratase 3 | 0.7072/1/1 |  | 5.9696/2/2 | 1.5852/4/4 |
| HAKAI_HUMAN | E3 ubiquitin-protein ligase Hakai | 0.3351/1/1 |  |  | 8.036/1/1 |
| HAUS4_HUMAN | HAUS augmin-like complex subunit 4 | 0.0423/1/1 |  |  | 57.61/1/1 |
| HCFC1_HUMAN | Host cell factor 1 | 0.5818/14/15 |  | 2.1768/1/1 |  |
| HDAC1_HUMAN | Histone deacetylase 1 | 0.504/6/6 |  |  | 1.694/1/2 |
| HMGN5_HUMAN | High mobility group nucleosome-binding domain-containing protein 5 | 0.5022/3/5 |  |  | 1.281/1/1 |
| HMOX2_HUMAN | Heme oxygenase 2 | 0.2554/1/1 |  |  | 2.454/1/1 |
| HNRPK_HUMAN | Heterogeneous nuclear ribonucleoprotein K | 0.6974/18/20 | 0.3529/8/8 | 2.0237/13/13 |  |
| HNRPL_HUMAN | Heterogeneous nuclear ribonucleoprotein L | 0.4883/15/18 | 0.4397/4/4 | 4.6695/3/3 | 3.1403/6/6 |
| HNRPM_HUMAN | Heterogeneous nuclear ribonucleoprotein M | 0.1427/22/22 | 0.0963/10/10 | 3.1112/2/2 | 1.8638/9/9 |
| HNRPR_HUMAN | Heterogeneous nuclear ribonucleoprotein R |  | 0.414/6/6 | 2.5436/7/7 | 1.7018/7/9 |
| HS12A_HUMAN | Heat shock 70 kDa protein 12A |  | 0.3411/1/1 |  | 162.5/1/1 |
| HS71L_HUMAN | Heat shock 70 kDa protein 1-like | 0.7175/5/9 |  | 2.9845/6/12 |  |
| IKIP_HUMAN | Inhibitor of nuclear factor kappa-B kinase-interacting protein | 0.0036/1/1 |  |  | 1.28/1/1 |
| ILVBL_HUMAN | Acetolactate synthase-like protein | 0.7452/1/1 |  |  | 1.7368/5/5 |
| ISY1_HUMAN | Pre-mRNA-splicing factor ISY1 homolog | 0.7378/3/3 | 0.5365/2/2 |  | 1.2502/2/2 |
| ITPR2_HUMAN | Inositol 1,4,5-trisphosphate receptor type 2 | 0.7227/3/3 |  | 6.085/1/1 | 1.3984/4/5 |
| ITPR3_HUMAN | Inositol 1,4,5-trisphosphate receptor type 3 | 0.6375/11/11 |  | 6.085/1/1 | 1.3919/5/7 |
| IWS1_HUMAN | Protein IWS1 homolog | 0.2619/2/2 |  |  | 1.1899/1/2 |
| JPH1_HUMAN | Junctophilin-1 | 0.0014/1/1 |  |  | 1.187/1/1 |
| KANL1_HUMAN | KAT8 regulatory NSL complex subunit 1 |  | 0.0941/1/1 | 50.79/1/1 |  |
| KDIS_HUMAN | Kinase D-interacting substrate of 220 kDa | 0.1464/1/1 |  |  | 1.1857/2/2 |
| KHDR1_HUMAN | KH domain-containing, RNA-binding, signal transduction-associated protein 1 | 0.3958/9/10 |  |  | 1.4948/3/3 |
| KI20A_HUMAN | Kinesin-like protein KIF20A | 0.5269/4/6 |  |  | 39.88/1/1 |
| KI67_HUMAN | Antigen KI-67 |  | 0.5479/19/21 |  | 2.0034/3/6 |
| L2GL2_HUMAN | Lethal(2) giant larvae protein homolog 2 | 0.0454/1/1 |  | 3.2906/2/2 | 1.1879/2/2 |
| LAMB1_HUMAN | Laminin subunit beta-1 |  | 0/1/1 |  | 1.3429/1/1 |
| LAP2A_HUMAN | Lamina-associated polypeptide 2, isoform alpha | 0.6326/10/16 |  | 4.0755/3/3 | 2.0451/2/3 |
| LAS1L_HUMAN | Ribosomal biogenesis protein LAS1L | 0.7405/3/7 |  |  | 1.6753/1/1 |
| LBR_HUMAN | Lamin-B receptor | 0.5617/6/6 | 0.071/1/1 |  | 2.069/1/1 |
| LCA5L_HUMAN | Lebercilin-like protein | 0.0248/1/1 |  |  | 6100000000/1/1 |
| LETM1_HUMAN | LETM1 and EF-hand domain-containing protein 1, mitochondrial | 0.3333/2/3 |  | 15.45/1/1 | 9.1652/2/2 |
| LIMA1_HUMAN | LIM domain and actin-binding protein 1 | 0.4822/14/16 | 0.0364/3/3 | 2.6793/2/3 |  |
| LMBD2_HUMAN | LMBR1 domain-containing protein 2 |  | 0.011/1/1 |  | 1.279/1/1 |
| LYAR_HUMAN | Cell growth-regulating nucleolar protein | 0.7084/4/4 | 0.4792/2/2 |  | 2.723/3/3 |
| LYRIC_HUMAN | Protein LYRIC | 0.6414/2/5 |  | 7.173/1/1 | 1.514/4/5 |
| MACD1_HUMAN | O-acetyl-ADP-ribose deacetylase MACROD1 |  | 0.452/2/2 |  | 2.1598/1/1 |
| MATR3_HUMAN | Matrin-3 | 0.2173/22/22 | 0.27/8/9 | 4.1396/3/3 | 2.0742/12/12 |
| MCE1_HUMAN | mRNA-capping enzyme |  | 0/1/1 |  | 3.036/1/1 |
| MDN1_HUMAN | Midasin | 0.684/2/8 |  |  | 1.5617/1/5 |
| MESD_HUMAN | LDLR chaperone MESD | 0.1304/1/2 |  | 2.876/1/1 |  |
| MINT_HUMAN | Msx2-interacting protein | 0.5245/3/11 | 0.5314/4/5 |  | 2.084/1/1 |
| MS18A_HUMAN | Protein Mis18-alpha | 0.2266/1/1 |  |  | 1.439/1/1 |
| MTA2_HUMAN | Metastasis-associated protein MTA2 | 0.5761/14/16 | 0.3655/5/5 | 353.4703/4/4 | 1.7301/3/5 |
| MTA3_HUMAN | Metastasis-associated protein MTA3 | 0.5277/6/6 | 0.3368/1/1 | 2.428/1/1 | 2.876/1/1 |
| MYH14_HUMAN | Myosin-14 | 0.223/2/2 |  |  | 1.3102/3/4 |
| MYO1C_HUMAN | Unconventional myosin-Ic | 0.249/13/13 |  | 8.3738/6/6 |  |
| MYO6_HUMAN | Unconventional myosin-VI | 0.1233/4/4 |  |  | 2.9159/4/5 |
| NCOA5_HUMAN | Nuclear receptor coactivator 5 | 0.4769/6/7 | 0.5738/3/3 |  | 2.0237/1/1 |
| NEB1_HUMAN | Neurabin-1 | 0.2712/2/2 |  |  | 1.287/1/1 |
| NECP2_HUMAN | Adaptin ear-binding coat-associated protein 2 | 0.5283/1/1 |  | 2.869/1/1 |  |
| NLE1_HUMAN | Notchless protein homolog 1 |  | 0.4597/1/1 |  | 1.4582/1/2 |
| NOLC1_HUMAN | Nucleolar and coiled-body phosphoprotein 1 |  | 0.5708/5/5 |  | 1.9169/1/1 |
| NONO_HUMAN | Non-POU domain-containing octamer-binding protein | 0.2521/20/21 | 0.144/8/9 | 5.015/2/2 | 1.9679/5/5 |
| NOP58_HUMAN | Nucleolar protein 58 |  | 0.2477/5/6 |  | 1.762/3/3 |
| NU205_HUMAN | Nuclear pore complex protein Nup205 |  | 0.3089/3/3 |  | 1.4282/3/3 |
| NUP50_HUMAN | Nuclear pore complex protein Nup50 | 0.5979/9/11 | 0.3688/2/2 |  | 1.4983/4/5 |
| NUSAP_HUMAN | Nucleolar and spindle-associated protein 1 |  | 0.5057/1/1 |  | 1.9285/2/4 |
| OAT_HUMAN | Ornithine aminotransferase, mitochondrial | 0.1563/1/3 |  |  | 2.9896/4/4 |
| ODR4_HUMAN | Protein odr-4 homolog | 0.7305/1/1 |  |  | 1.3468/1/1 |
| OGT1_HUMAN | UDP-N-acetylglucosamine--peptide N-acetylglucosaminyltransferase 110 kDa subunit | 0.5653/2/2 | 0.5655/1/1 | 47.43/1/1 | 26.1288/2/7 |
| P66A_HUMAN | Transcriptional repressor p66-alpha | 0.3862/7/7 | 0.5681/2/2 |  | 2.039/1/1 |
| P66B_HUMAN | Transcriptional repressor p66-beta |  | 0.4723/2/2 |  | 1.2926/1/1 |
| PA24A_HUMAN | Cytosolic phospholipase A2 | 0.3419/3/3 |  | 13.4155/2/2 |  |
| PABP2_HUMAN | Polyadenylate-binding protein 2 | 0.5335/7/7 | 0.2987/2/2 | 2.481/1/1 | 1.1887/3/3 |
| PALLD_HUMAN | Palladin | 0.2484/4/4 | 0.245/1/1 |  | 1.2619/3/3 |
| PEO1_HUMAN | Twinkle protein, mitochondrial | 0.4996/2/2 |  |  | 2.743/1/1 |
| PEPL_HUMAN | Periplakin | 0.2069/9/9 |  |  | 1.5202/2/3 |
| PERI_HUMAN | Peripherin |  | 0.024/1/2 | 2.3328/2/2 | 1.5297/1/1 |
| PHC2_HUMAN | Polyhomeotic-like protein 2 | 0.694/1/2 |  |  | 1.316/1/1 |
| PHF14_HUMAN | PHD finger protein 14 | 0.3017/5/8 |  |  | 3.579/1/1 |
| PHF3_HUMAN | PHD finger protein 3 | 0.6633/7/8 |  |  | 1.7016/1/2 |
| PININ_HUMAN | Pinin |  | 0.3885/3/3 | 2.962/1/1 | 2.576/1/1 |
| PLD3_HUMAN | Phospholipase D3 | 0.0074/1/1 |  | 550.4/1/1 |  |
| PLEC_HUMAN | Plectin | 0.2751/190/196 | 0.1086/72/74 | 4.4278/42/47 | 2.7159/77/79 |
| PLOD1_HUMAN | Procollagen-lysine,2-oxoglutarate 5-dioxygenase 1 |  | 0.5246/2/2 | 32.0162/4/4 |  |
| PLOD2_HUMAN | Procollagen-lysine,2-oxoglutarate 5-dioxygenase 2 | 0.7113/2/7 |  | 4.0662/2/2 |  |
| PLOD3_HUMAN | Procollagen-lysine,2-oxoglutarate 5-dioxygenase 3 | 0.5699/10/11 | 0.4759/2/2 | 3.154/5/5 | 1.1828/8/11 |
| PLST_HUMAN | Plastin-3 | 0.5812/2/2 |  | 2.2255/6/9 |  |
| PP1RA_HUMAN | Serine/threonine-protein phosphatase 1 regulatory subunit 10 | 0.4128/7/7 |  |  | 1.2322/1/2 |
| PR40A_HUMAN | Pre-mRNA-processing factor 40 homolog A | 0.7428/6/11 | 0.5401/2/2 |  | 1.818/1/1 |
| PRDBP_HUMAN | Protein kinase C delta-binding protein | 0.4753/1/1 |  | 2.256/1/1 | 2.8853/2/2 |
| PRP17_HUMAN | Pre-mRNA-processing factor 17 | 0.6081/6/6 |  | 7.224/1/1 |  |
| PRP8_HUMAN | Pre-mRNA-processing-splicing factor 8 | 0.6147/30/45 |  |  | 1.3645/29/30 |
| PSIP1_HUMAN | PC4 and SFRS1-interacting protein |  | 0.4043/7/7 |  | 15.57/1/1 |
| PSPC1_HUMAN | Paraspeckle component 1 | 0.4501/10/12 | 0.2438/1/1 | 9.3991/3/3 | 2.0014/2/2 |
| PTBP1_HUMAN | Polypyrimidine tract-binding protein 1 | 0.3694/17/18 |  | 3.8305/8/8 |  |
| PTBP3_HUMAN | Polypyrimidine tract-binding protein 3 | 0.4766/4/6 |  | 230.0245/2/2 |  |
| PTPRB_HUMAN | Receptor-type tyrosine-protein phosphatase beta |  | 0/1/1 | 33.07/1/1 |  |
| PUR2_HUMAN | Trifunctional purine biosynthetic protein adenosine-3 | 0.6698/4/4 |  | 2.2445/10/12 |  |
| PWP2_HUMAN | Periodic tryptophan protein 2 homolog |  | 0.2552/4/4 |  | 4.76/1/1 |
| PXDN_HUMAN | Peroxidasin homolog | 0.7407/1/1 |  |  | 1.2256/1/1 |
| QSOX2_HUMAN | Sulfhydryl oxidase 2 | 0.0009/1/1 |  |  | 3.205/1/1 |
| RAE1L_HUMAN | mRNA export factor |  | 0.193/1/1 | 2.406/1/1 |  |
| RAGP1_HUMAN | Ran GTPase-activating protein 1 | 0.5214/13/15 |  | 2.3872/3/3 | 1.3636/6/6 |
| RALY_HUMAN | RNA-binding protein Raly |  | 0.5137/2/2 | 1/1/5133 | 2.03/1/1 |
| RBM14_HUMAN | RNA-binding protein 14 | 0.2211/13/13 | 0.0798/1/1 |  | 1.6308/2/2 |
| RBM15_HUMAN | Putative RNA-binding protein 15 | 0.735/8/13 | 0.4961/3/3 |  | 1.4192/6/6 |
| RBM25_HUMAN | RNA-binding protein 25 | 0.6573/9/13 |  |  | 2.7275/4/4 |
| RBM28_HUMAN | RNA-binding protein 28 |  | 0.1884/5/5 |  | 1.9047/1/1 |
| RBM33_HUMAN | RNA-binding protein 33 | 0.7447/1/1 | 0.0006/2/2 |  | 204.7744/2/2 |
| RBMX_HUMAN | RNA-binding motif protein, X chromosome | 0.7064/13/15 | 0.285/1/1 | 6.884/1/1 | 3.2891/5/5 |
| RBP2_HUMAN | E3 SUMO-protein ligase RanBP2 | 0.4485/31/38 | 0.3809/9/10 | 12.0081/4/4 | 1.5007/12/13 |
| REPS1_HUMAN | RalBP1-associated Eps domain-containing protein 1 |  | 0/1/1 | 2.191/1/1 |  |
| RGPD8_HUMAN | RANBP2-like and GRIP domain-containing protein 8 | 0.6834/4/6 |  |  | 2.1672/2/2 |
| RING1_HUMAN | E3 ubiquitin-protein ligase RING1 | 0.5252/4/4 |  |  | 1.285/1/1 |
| RL38_HUMAN | 60S ribosomal protein L38 | 0.0539/2/2 |  | 4.032/2/2 | 1.761/1/1 |
| RLF_HUMAN | Zinc finger protein Rlf | 0.1473/2/3 |  |  | 1.407/1/1 |
| RNPS1_HUMAN | RNA-binding protein with serine-rich domain 1 | 0.5421/3/6 |  |  | 1.714/1/1 |
| ROA0_HUMAN | Heterogeneous nuclear ribonucleoprotein A0 | 0.6212/9/11 |  | 3.2755/5/5 |  |
| ROA1_HUMAN | Heterogeneous nuclear ribonucleoprotein A1 | 0.5264/14/15 | 0.0832/6/6 | 4.5871/5/5 | 1.3267/8/10 |
| ROA2_HUMAN | Heterogeneous nuclear ribonucleoproteins A2/B1 | 0.543/17/19 | 0.3519/8/10 | 2.9374/7/7 | 2.2725/11/11 |
| RPA1_HUMAN | DNA-directed RNA polymerase I subunit RPA1 |  | 0.5122/6/7 |  | 3.0331/3/3 |
| RPA2_HUMAN | DNA-directed RNA polymerase I subunit RPA2 | 0.5037/4/5 | 0.397/2/2 |  | 2.677/1/1 |
| RPB3_HUMAN | DNA-directed RNA polymerase II subunit RPB3 | 0.4661/2/3 | 0.2495/1/1 |  | 1.2223/3/3 |
| RPR1A_HUMAN | Regulation of nuclear pre-mRNA domain-containing protein 1A | 0.4859/1/1 |  |  | 5.688/1/1 |
| RPR1B_HUMAN | Regulation of nuclear pre-mRNA domain-containing protein 1B | 0.1918/5/5 | 0.1668/2/2 | 2.566/1/1 |  |
| RPRD2_HUMAN | Regulation of nuclear pre-mRNA domain-containing protein 2 | 0.4327/7/9 | 0.2623/1/1 | 4.823/1/1 |  |
| RRP12_HUMAN | RRP12-like protein |  | 0.5659/6/8 | 2.2896/1/1 | 1.7852/8/8 |
| RSBN1_HUMAN | Round spermatid basic protein 1 | 0.3442/5/5 | 0.0824/1/1 | 923.3/1/1 |  |
| RT05_HUMAN | 28S ribosomal protein S5, mitochondrial | 0.1371/1/1 |  |  | 4.8673/2/2 |
| RT11_HUMAN | 28S ribosomal protein S11, mitochondrial | 0.1581/1/1 |  |  | 2.3505/1/1 |
| RU17_HUMAN | U1 small nuclear ribonucleoprotein 70 kDa |  | 0.3888/4/4 | 3.381/1/1 | 1.203/4/5 |
| RU1C_HUMAN | U1 small nuclear ribonucleoprotein C | 0.3619/1/1 |  | 2.134/1/1 |  |
| RU2A_HUMAN | U2 small nuclear ribonucleoprotein A' | 0.6723/7/8 | 0.5741/4/4 |  | 1.7117/6/6 |
| RUXF_HUMAN | Small nuclear ribonucleoprotein F | 0.577/2/2 | 0.2633/1/1 | 2.1112/1/1 |  |
| RUXGL_HUMAN | Putative small nuclear ribonucleoprotein G-like protein 15 | 0.4802/2/2 | 0.4144/1/1 |  | 1.2576/2/2 |
| S10AA_HUMAN | Protein S100-A10 | 0.2157/1/1 | 0.1809/1/1 | 4.212/1/1 |  |
| S38AA_HUMAN | Putative sodium-coupled neutral amino acid transporter 10 | 0.0033/1/1 |  |  | 1.69/1/1 |
| SAFB1_HUMAN | Scaffold attachment factor B1 | 0.5182/15/17 | 0.2757/6/6 | 6.238/4/4 | 3.6845/4/4 |
| SAFB2_HUMAN | Scaffold attachment factor B2 |  | 0.3037/7/7 | 5.1625/2/2 | 3.5091/3/3 |
| SEPT2_HUMAN | Septin-2 | 0.74/9/12 |  | 3.0232/4/4 |  |
| SEPT6_HUMAN | Septin-6 | 0.6843/4/4 |  | 2.2522/2/2 | 1.1818/2/2 |
| SEPT7_HUMAN | Septin-7 | 0.633/8/8 |  | 3.2879/6/6 |  |
| SEPT9_HUMAN | Septin-9 | 0.6608/12/15 |  | 1.9852/2/2 |  |
| SF3A1_HUMAN | Splicing factor 3A subunit 1 | 0.5655/11/19 | 0.4464/8/9 | 3.1441/1/1 | 1.3703/11/12 |
| SF3B1_HUMAN | Splicing factor 3B subunit 1 | 0.7293/23/31 | 0.5508/11/11 | 5.4408/3/3 | 1.9732/24/24 |
| SF3B3_HUMAN | Splicing factor 3B subunit 3 |  | 0.4651/5/5 |  | 1.1912/15/16 |
| SF3B5_HUMAN | Splicing factor 3B subunit 5 |  | 0.2511/1/1 |  | 1.364/1/1 |
| SF3B6_HUMAN | Splicing factor 3B subunit 6 | 0.2043/3/3 |  |  | 2.2772/3/3 |
| SFPQ_HUMAN | Splicing factor, proline- and glutamine-rich | 0.372/26/26 | 0.0761/6/6 | 6.0995/8/8 | 2.5987/7/7 |
| SIN3A_HUMAN | Paired amphipathic helix protein Sin3a | 0.4538/10/10 |  |  | 1.503/1/1 |
| SK2L2_HUMAN | Superkiller viralicidic activity 2-like 2 | 0.6922/9/11 |  | 2.1144/2/2 |  |
| SKAP_HUMAN | Small kinetochore-associated protein | 0.0016/1/1 | 0.1693/1/1 |  | 73.28/1/1 |
| SMD1_HUMAN | Small nuclear ribonucleoprotein Sm D1 | 0.224/3/3 |  | 14952.4839/1/1 |  |
| SMRC1_HUMAN | SWI/SNF complex subunit SMARCC1 |  | 0.4367/4/5 |  | 1.832/1/1 |
| SMRC2_HUMAN | SWI/SNF complex subunit SMARCC2 |  | 0.476/5/5 |  | 1.628/3/3 |
| SNR27_HUMAN | U4/U6.U5 small nuclear ribonucleoprotein 27 kDa protein | 0.6367/2/3 |  |  | 1.559/1/1 |
| SNR40_HUMAN | U5 small nuclear ribonucleoprotein 40 kDa protein | 0.3625/5/7 | 0.4333/1/1 |  | 1.4535/4/4 |
| SNW1_HUMAN | SNW domain-containing protein 1 |  | 0.1738/1/1 |  | 18.4584/2/4 |
| SON_HUMAN | Protein SON | 0.5085/20/22 | 0.1981/4/5 |  | 7.13/1/1 |
| SPAS2_HUMAN | Spermatogenesis-associated serine-rich protein 2 | 0.4502/2/2 |  |  | 1.228/1/1 |
| SPF27_HUMAN | Pre-mRNA-splicing factor SPF27 |  | 0.4125/3/3 | 2.997/1/1 |  |
| SPT6H_HUMAN | Transcription elongation factor SPT6 | 0.1824/20/20 | 0.3019/5/5 | 6.2192/3/3 | 1.2091/12/17 |
| SPTB2_HUMAN | Spectrin beta chain, non-erythrocytic 1 | 0.1666/54/55 | 0.095/29/32 | 2.8963/15/15 |  |
| SPTN1_HUMAN | Spectrin alpha chain, non-erythrocytic 1 | 0.1479/82/83 | 0.0281/47/47 | 2.3006/26/28 | 1.4219/33/36 |
| SPTN2_HUMAN | Spectrin beta chain, non-erythrocytic 2 | 0.2107/17/17 | 0.0779/9/9 |  | 1.8848/8/8 |
| SR140_HUMAN | U2 snRNP-associated SURP motif-containing protein | 0.7179/5/6 | 0.4949/3/3 |  | 1.6025/5/5 |
| SRRM2_HUMAN | Serine/arginine repetitive matrix protein 2 | 0.5706/29/31 | 0.1747/2/2 |  | 1.5048/6/7 |
| SRSF6_HUMAN | Serine/arginine-rich splicing factor 6 | 0.5194/11/12 | 0.4093/3/3 |  | 1.3997/1/2 |
| SRSF7_HUMAN | Serine/arginine-rich splicing factor 7 |  | 0.5717/2/2 |  | 1.3605/4/4 |
| SUMO1_HUMAN | Small ubiquitin-related modifier 1 | 0.4085/1/1 |  | 2.798/1/1 |  |
| SURF4_HUMAN | Surfeit locus protein 4 | 0.0254/1/2 |  |  | 1.1854/3/3 |
| SYF1_HUMAN | Pre-mRNA-splicing factor SYF1 | 0.6762/7/7 | 0.3475/4/4 |  | 1.4683/7/7 |
| SYMPK_HUMAN | Symplekin | 0.6877/4/4 | 0.2639/1/1 | 2.2432/1/1 | 1.6576/4/4 |
| SYNE2_HUMAN | Nesprin-2 | 0.4411/8/12 | 0.3828/2/2 | 2.539/1/1 | 1.42/1/1 |
| TADBP_HUMAN | TAR DNA-binding protein 43 | 0.5841/6/6 |  | 2.437/2/2 |  |
| TBL3_HUMAN | Transducin beta-like protein 3 |  | 0.5119/5/6 |  | 1.4917/4/4 |
| TCOF_HUMAN | Treacle protein |  | 0.4322/10/10 |  | 1.2528/3/4 |
| TDIF2_HUMAN | Deoxynucleotidyltransferase terminal-interacting protein 2 |  | 0.576/3/3 |  | 1.4828/2/2 |
| TGON2_HUMAN | Trans-Golgi network integral membrane protein 2 |  | 0.3661/1/1 |  | 1.295/1/1 |
| THIL_HUMAN | Acetyl-CoA acetyltransferase, mitochondrial |  | 0.4333/1/2 | 2.576/1/1 | 3.3883/5/5 |
| TIM13_HUMAN | Mitochondrial import inner membrane translocase subunit Tim13 | 0.0354/1/1 |  |  | 1.7711/1/1 |
| TIM8A_HUMAN | Mitochondrial import inner membrane translocase subunit Tim8 A | 0.6545/1/1 |  |  | 1.739/1/1 |
| TM165_HUMAN | Transmembrane protein 165 | 0.0001/1/1 |  |  | 1.917/1/1 |
| TM256_HUMAN | Transmembrane protein 256 | 0.2387/1/1 |  |  | 4.9234/1/1 |
| TOP3A_HUMAN | DNA topoisomerase 3-alpha | 0.6039/2/3 | 0.2948/1/1 |  | 1.48/1/1 |
| TP53B_HUMAN | Tumor suppressor p53-binding protein 1 |  | 0.5765/5/5 |  | 1.2548/1/2 |
| TPR_HUMAN | Nucleoprotein TPR |  | 0.5281/15/17 |  | 1.5198/16/17 |
| TR150_HUMAN | Thyroid hormone receptor-associated protein 3 | 0.5188/17/19 | 0.3023/5/6 |  | 2.0865/3/3 |
| TRIO_HUMAN | Triple functional domain protein |  | 0.002/2/2 |  | 1.1717/1/2 |
| TSG10_HUMAN | Testis-specific gene 10 protein | 0.2521/1/1 |  |  | 28100000000/1/1 |
| U2AF1_HUMAN | Splicing factor U2AF 35 kDa subunit | 0.7001/1/2 |  | 2.025/1/2 |  |
| UHRF1_HUMAN | E3 ubiquitin-protein ligase UHRF1 | 0.6272/17/19 |  |  | 1.2873/2/2 |
| UTRO_HUMAN | Utrophin | 0.1949/1/1 |  |  | 1.2501/4/5 |
| VDR_HUMAN | Vitamin D3 receptor | 0.7047/1/1 |  |  | 18.37/1/1 |
| VIME_HUMAN | Vimentin |  | 0.1975/6/8 | 2.9181/24/24 | 1.601/28/30 |
| WDR1_HUMAN | WD repeat-containing protein 1 | 0.4185/2/2 |  | 2.0342/7/7 |  |
| WDR33_HUMAN | pre-mRNA 3' end processing protein WDR33 | 0.4458/5/6 | 0.2841/1/1 |  | 4.767/1/1 |
| WDR61_HUMAN | WD repeat-containing protein 61 | 0.069/2/2 |  | 3.0835/4/4 |  |
| YKT6_HUMAN | Synaptobrevin homolog YKT6 | 0.5085/2/2 |  | 3543.022/2/2 |  |
| ZCH18_HUMAN | Zinc finger CCCH domain-containing protein 18 | 0.5692/9/9 | 0.2302/2/2 |  | 2.1186/5/5 |
| ZFR_HUMAN | Zinc finger RNA-binding protein | 0.3949/10/12 | 0.2806/3/3 |  | 2.3523/3/3 |
| ZN106_HUMAN | Zinc finger protein 106 | 0.0177/5/5 | 0.4239/1/1 |  | 2.728/1/1 |
| ZN292_HUMAN | Zinc finger protein 292 |  | 0.0003/1/1 | 64.42/1/1 |  |
| ZN687_HUMAN | Zinc finger protein 687 | 0.1434/2/2 | 0.2096/1/1 |  | 1.919/1/1 |
| ZNRF2_HUMAN | E3 ubiquitin-protein ligase ZNRF2 |  | 0.0072/1/1 |  | 1.8081/1/1 |
| ZO2_HUMAN | Tight junction protein ZO-2 | 0.2389/2/2 |  |  | 1.3485/1/4 |
